# Inhibiting translation elongation can aid genome duplication in *Escherichia coli*

**DOI:** 10.1093/nar/gkw1254

**Published:** 2016-12-12

**Authors:** Kamila K. Myka, Michelle Hawkins, Aisha H. Syeda, Milind K. Gupta, Caroline Meharg, Mark S. Dillingham, Nigel J. Savery, Robert G. Lloyd, Peter McGlynn

**Affiliations:** 1 Department of Biology, University of York, Wentworth Way, York YO10 5DD, UK; 2 School of Medical Sciences, Institute of Medical Sciences, University of Aberdeen, Aberdeen AB25 2ZD, UK; 3 Institute for Global Food Security, Queen's University Belfast, David Keir Building, Malone Road, Belfast BT9 5BN, UK; 4 DNA-Protein Interactions Unit, School of Biochemistry, University of Bristol, Bristol BS8, 1TD, UK; 5 Centre for Genetics and Genomics, University of Nottingham, Queen's Medical Centre, Nottingham NG7 2UH, UK

## Abstract

Conflicts between replication and transcription challenge chromosome duplication. *Escherichia coli* replisome movement along transcribed DNA is promoted by Rep and UvrD accessory helicases with Δ*rep* Δ*uvrD* cells being inviable under rapid growth conditions. We have discovered that mutations in a tRNA gene, *aspT*, in an aminoacyl tRNA synthetase, AspRS, and in a translation factor needed for efficient proline–proline bond formation, EF-P, suppress Δ*rep* Δ*uvrD* lethality. Thus replication-transcription conflicts can be alleviated by the partial sacrifice of a mechanism that reduces replicative barriers, namely translating ribosomes that reduce RNA polymerase backtracking. Suppression depends on RelA-directed synthesis of (p)ppGpp, a signalling molecule that reduces replication-transcription conflicts, with RelA activation requiring ribosomal pausing. Levels of (p)ppGpp in these suppressors also correlate inversely with the need for Rho activity, an RNA translocase that can bind to emerging transcripts and displace transcription complexes. These data illustrate the fine balance between different mechanisms in facilitating gene expression and genome duplication and demonstrate that accessory helicases are a major determinant of this balance. This balance is also critical for other aspects of bacterial survival: the mutations identified here increase persistence indicating that similar mutations could arise in naturally occurring bacterial populations facing antibiotic challenge.

## INTRODUCTION

Cell survival from one generation to the next relies on efficient and faithful replication of the genome. However, the template for replication frequently harbours obstacles that have the potential to interfere with the progression of replisome complexes, the macromolecular machines responsible for driving genome duplication. Protein–DNA complexes are important sources of such obstacles and those associated with transcription are especially problematic, in part because of their abundance and in part because they present multiple different challenges to replisome movement (1,2). One major challenge is the very high free energy of binding of transcription complexes that creates a need to disrupt many RNA polymerase–nucleic acid interactions as replication proceeds. Transcribing RNA polymerases (RNAPs) also pause frequently either spontaneously or upon encountering DNA template damage (3). Paused RNAPs can also diffuse backwards along the template resulting in displacement of the transcript 3΄ end from the RNAP active site. This causes an inability to resume transcription, creating barriers to replication that threaten genome stability (4,5).

Mechanisms exist that reduce the probability of a replication fork encountering paused transcription complexes. Upstream RNAPs on the DNA and ribosomes on the emerging transcript may inhibit backtracking of a paused transcription complex in bacteria (5–7). RNA translocases such as the bacterial Rho hexamer can also translocate along transcripts not coated with ribosomes and displace paused and blocked RNAPs (5,8,9). Rho translocation also disrupts R-loops, structures in which an RNA transcript hybridizes to the DNA template strand (9). Nucleases can also aid genome duplication by promoting the resumption of transcription by backtracked RNAP (5,10). GreA and GreB bind to *E. coli* RNAP and stimulate cleavage of backtracked transcripts, creating a new RNA 3΄ end that can be used to restart transcription (11). Conversely, the 3΄-5΄ helicase UvrD can increase rather than decrease backtracking of RNAP and this may increase access of nucleotide excision repair enzymes UvrABC to DNA damage thus helping to clear barriers to RNAP and aid genome duplication (12).

Signalling molecules such as guanosine tetraphosphate and pentaphosphate in bacteria (referred to collectively as (p)ppGpp) can also reprogramme transcription. (p)ppGpp is central to the stringent response in bacteria, identified originally as a response to amino acid starvation in which the presence of uncharged tRNA within the ribosomal A site stimulates ribosome-bound RelA to synthesize (p)ppGpp (13–15). Control of the stringent response also requires a (p)ppGpp-specific pyrophosphohydrolase, SpoT, that also has low (p)ppGpp synthase activity (15). However, the synthase activity of SpoT is induced by other stresses such as depletion of fatty acids (16). Central to the stringent response is binding of (p)ppGpp to RNAP which leads to downregulation of stable RNA production and upregulation of stress response genes (15,17,18). Expression of ribosomal rRNA operons accounts for half of all transcription during rapid growth when (p)ppGpp levels are low and thus provides the majority of transcriptional obstacles to replisomes *in vivo* when cells are dividing quickly (19–22). Elevated (p)ppGpp therefore reduces conflicts between replication and transcription by decreasing the density of transcribing RNAPs on the genome. (p)ppGpp can also decrease the stability of transcription complexes blocked by template damage *in vitro* which may decrease the density of blocked RNAP on the genome (10). This destabilization has been questioned, though, and a role for (p)ppGpp in increasing the fidelity of transcription and hence reducing RNAP pausing and backtracking has been proposed (23). Other work implicates (p)ppGpp acting synergistically with UvrD in promoting backtracking of paused RNAP, one function of which could be to facilitate transcription-coupled repair of any pause-inducing DNA damage (24). Thus, how (p)ppGpp might act on transcription elongation complexes, as opposed to transcription initiation, is still far from clear. Transcription is not the only target of (p)ppGpp, though, since (p)ppGpp also binds multiple other targets. Inhibited targets include DnaG primase (25–28) and the translation elongation factors EFG and EF-Tu (29), indicating the pleiotropic impact of elevated (p)ppGpp on replication, transcription and elongation. (p)ppGpp also affects other diverse aspects of metabolism such as phospholipid synthesis, oxidative metabolism and resistance to antibiotics (30). These signalling molecules also enhance the ability of *E. coli* to persist in the presence of antibiotics. Persistence is the non-heritable ability of a small fraction of a bacterial population to survive exposure to an otherwise lethal concentration of antibiotic (31). (p)ppGpp levels vary stochastically in a bacterial population and increased (p)ppGpp activates toxin–antitoxin systems that induce slow growth in a sub-population of cells, leading to antibiotic tolerance (32).

Mechanisms also exist to increase the probability of continued replisome movement in the event of a collision with an RNAP. Accessory replicative motors provide a supply of additional helicases at the fork to aid protein displacement ahead of the replisome (33–37). In *E. coli* Rep helicase promotes movement of replisomes along protein-bound DNA *in vitro* and *in vivo* (20,34). Rep appears to be the main replisome anti-pausing factor in *E. coli* (38) and absence of Rep results in at least a two-fold increase in genome duplication time (39,40) and higher dependence on recombination-directed repair of damaged forks (41–44). However, Δ*rep* cells remain viable since the homologous helicase UvrD can substitute for the absence of Rep at the replication fork (20,34). This substitution is only partial, though, and correlates with a physical and functional interaction between DnaB and Rep but not between DnaB and UvrD (34,45). This partial functional overlap is sufficient for single deletion mutants to be viable during rapid growth whereas Δ*rep* Δ*uvrD* cells are not (46). Δ*rep* Δ*uvrD* inviability can be suppressed by growth on minimal medium, conditions under which (p)ppGpp levels are high, and also by elevation of (p)ppGpp levels on rich medium via the *spoT1* mutation encoding a (p)ppGpp pyrophosphorylase-defective SpoT (20,34). Mutations within *rpo* genes can also suppress Δ*rep* Δ*uvrD* rich medium lethality (20,34,47). These mutant RNAPs display different phenotypes suggesting distinct mechanisms of suppression but some seem to phenocopy elevated (p)ppGpp and/or reduce RNAP backtracking (10,47,48).

The above mechanisms reduce conflicts between replication and transcription but under rapid growth conditions forks are still blocked sufficiently frequently to require replisome reloading enzymes to maintain viability (49,50). Replisome reloading also often requires remodeling of the DNA at the blocked fork by recombination enzymes in order to generate a DNA structure suitable for replisome reloading (51). However, recombinational processing can lead to genome instability and is thus tightly controlled. One control is exerted by UvrD as it can disrupt RecA-ssDNA filaments and this disruption prevents excessive RecFOR-dependent loading of RecA onto ssDNA at blocked forks (52). Such excessive RecA loading contributes to Δ*rep* Δ*uvrD* lethality, evinced by the weak suppression of Δ*rep* Δ*uvrD* rich medium lethality via mutations in *recF, recO* or *recR* (34,53,54).

We have searched for suppressors of Δ*rep* Δ*uvrD* rich medium lethality that are not within *rpo, spoT, recF, O* or *R*. We identified a spontaneous suppressor in a tRNA gene, *aspT[t8c]*, that mutated a highly conserved residue that is structurally important in other tRNA species. Probing the basis of this suppression revealed that defective tRNA aminoacylation or inefficient peptide bond formation within the ribosome also suppressed Δ*rep* Δ*uvrD* lethality. In all cases, suppression required RelA-directed (p)ppGpp synthesis indicating that stalling of ribosome translocation by uncharged tRNA in the A site of ribosomes underpinned suppression. Thus, although efficient translation elongation aids genome duplication (5,7), the partial inhibition of ribosome translocation is more effective at mitigating replication-transcription conflicts. These data illustrate the fine balance between the multiple mechanisms that promote simultaneous gene expression and genome duplication and reveal the importance of accessory replicative helicase activity in determining this balance.

## MATERIALS AND METHODS

### Plasmids and strains

pAM403 (55) and pAM407 (34) are pRC7 derivatives encoding *rep* and *uvrD*, respectively. p3LC-TL30-5P and p3LC-TL30-5D contain a *cadC–lacZ* gene fusion in which five tandem codons present within the linker encode proline or aspartate, respectively (56). Strains were constructed by P1 *vir* transduction and are listed in Supplementary Table S1.

### Genome sequencing and analysis

DNA was extracted from 1 culture each of strain N7153 and N7182 grown in LB broth to stationary phase using Qiagen 100/G genomic tips from 5 ml cultures following the manufacturer's protocol. Genome sequencing was performed using an Illumina GAIIx instrument with 100 bp paired end reads. Paired reads were trimmed to remove adapters and mapped against the *E. coli* K12 strain MG1655 NC_000913 genome using bwa (57), duplicates marked using Picard (http://broadinstitute.github.io/picard) and variant analysis performed with SAMtools (58), followed by merging of variant tables using perl. Identified high quality synonymous and non-synonymous single nucleotide polymorphisms were annotated manually using the Integrative Genomics Viewer (59). The Illumina data were submitted in the form of fastq files to the European Nucleotide Archive (ENA) and are available under accession number PRJEB14483 at http://www.ebi.ac.uk/ena/data/view/PRJEB14483.

### Growth assays

All steps in plasmid loss assays were carried out at 37°C except those shown in Figures 2B and 3B in which all steps were conducted at 30°C or 25°C as indicated. Colonies were grown in LB broth and agar except the assays in Figure 2B and Supplementary Figures S3B and S4 which were performed with a defined rich medium broth and agar containing 0.2% glycerol (Figure 2B) or 0.2% glucose (Supplementary Figures S3B and S4) as a carbon source (60). The plates used in Supplementary Figures S3B and S4 also had decreasing concentrations of aspartate or phenylalanine, as indicated. Strains carrying derivatives of pRC7 were grown in LB broth or defined rich medium with 100 μg ml^−1^ of ampicillin overnight, diluted 100-fold into the same type of fresh liquid medium without ampicillin and grown to *A*_650_ 0.4. Dilutions were then plated onto LB or defined rich medium agar plates containing 120 μg ml^−1^ X-gal and 1 mM IPTG and incubated for 48 h except those shown in Figure 2B. Plates were then photographed and scored for blue/white colony formation.

For assays to assess colony forming ability, strains were grown in LB broth overnight at 37°C or, when temperature sensitive strains were involved, at 30°C. Serial 10-fold dilutions were made with 56/2 salts (61) on ice and then 5 μl of each dilution was spotted onto LB agar plates. Plates were then incubated at 37°C for 16 h unless otherwise stated. Ampicillin and bicyclomycin were included in LB agar plates where indicated at 100 and 25 μg ml^−1^, respectively.

For the colony formation assays in Supplementary Figure S3A, strains were grown in defined rich medium broth (60) containing 0.2% glucose, all amino acids and 100 μg ml^−1^ ampicillin overnight at 37°C. Tenfold serial dilutions were made in 56/2 salts and then 5 μl of each dilution spotted onto defined rich medium plates containing 0.2% glucose, 100 μg ml^−1^ ampicillin and with all amino acids or missing either aspartate or phenylalanine. These plates were incubated at 37°C for 16 h. Minimal medium agar plates (61) were used in Supplementary Figure S3C without and with 100 μg ml^−1^ ampicillin as indicated and incubated at 37°C for 72 h.

Colony-forming ability at increasing doses of UV light was assessed as described (62). Mismatch repair capacity was measured as the fraction of cells in a culture that acquired spontaneous mutations leading to rifampicin resistance. Briefly, overnight LB cultures were washed once in 56/2 salts and serially diluted 10-fold. 100 μl of the neat and the 10^−1^ dilution were spread on LB agar containing 15 μg ml^−1^ rifampicin. To estimate the total cfu ml^−1^ in the overnight culture, 10 μl of the 10^−6^ dilution was spotted in triplicate on LB agar without rifampicin. Plates were then incubated at 37°C for 16 h and the colonies then counted.

The liquid growth assays in Figure 2A were performed using a Tecan Infinite M200 Pro plate reader. Overnight LB cultures were diluted to *A*_600_ 0.005 in LB and 150 μl of each culture was transferred into 20 wells of a 96-well flat bottom plate (Corning). The microplate was incubated at 37°C for 24 h and *A*_600_ measurements were performed every 30 min preceded by plate shaking.

### Persistence

Single colonies were inoculated into 5 ml LB broth and grown with shaking at 37°C overnight. 50 μl of overnight culture was then inoculated into 5 ml of LB broth and grown at 37°C with shaking until 1–2 × 10^8^ colony forming units ml^−1^ reached. 500 μl was then transferred into two 1.5 ml microcentrifuge tubes. The first tube was centrifuged at 6000 rpm for 5 min at room temperature, resuspended in 500 μl 56/2 salts, serially diluted tenfold in 56/2 salts and 10 μl volumes of the 10^−4^ and 10^−5^ dilutions spotted onto LB agar plates containing 20 mM MgSO_4_ in triplicate. To the second tube 5 μl of 10 μg ml^−1^ ciprofloxacin was added and the tube was then inserted into a 50 ml Falcon tube and placed in a shaking incubator at 37°C for 5 h. After the 5 h incubation, this tube was centrifuged at 6000 rpm for 5 min at room temperature, resuspended in 500 μl 56/2 salts, recentrifuged and resuspended in 500 μl 56/2 salts. Colony-forming units were then assayed as for the first tube by serial dilution into 56/2 salts and plating in triplicate onto LB agar containing 20 mM MgSO_4_. MgSO_4_ was included to inhibit the activity of any traces of ciprofloxacin not removed by washing of the cells (63). All plates were incubated at 37°C overnight and then the fraction of colony forming units surviving ciprofloxacin exposure calculated.

### Flow cytometry

Analysis of chromosome content after completion of all ongoing rounds of replication, so-called run-out conditions, was performed on mid-log phase cultures after treatment with rifampicin and cephalexin as described (64) using a Becton Coulter CyAn ADP cytometer with 488 nm excitation and a 530/540 nm bandpass filter. Analysis of (p)ppGpp-dependent formation of RpoS-mCherry by flow cytometry was performed by growing the indicated strains overnight in LB, transferring 100 μl into 10 ml LB in a 125 ml flask followed by incubation at 37°C for 2 h. Then 1 ml of the culture was centrifuged, resuspended in 2 ml of 10% LB in M9 medium and analysed by flow cytometry on a Becton Dickinson LSRFortessa X-20 flow cytometer using 561 nm laser excitation and dection using a 610/620 nm bandpass filter.

### Translation assays

β-Galactosidase activity assays monitoring the relative levels of translation of *cadC*–*lacZ* fusions were performed in LB broth as described (56).

## RESULTS

### A tRNA mutation suppresses the requirement for accessory replicative helicases

**Figure 1.**
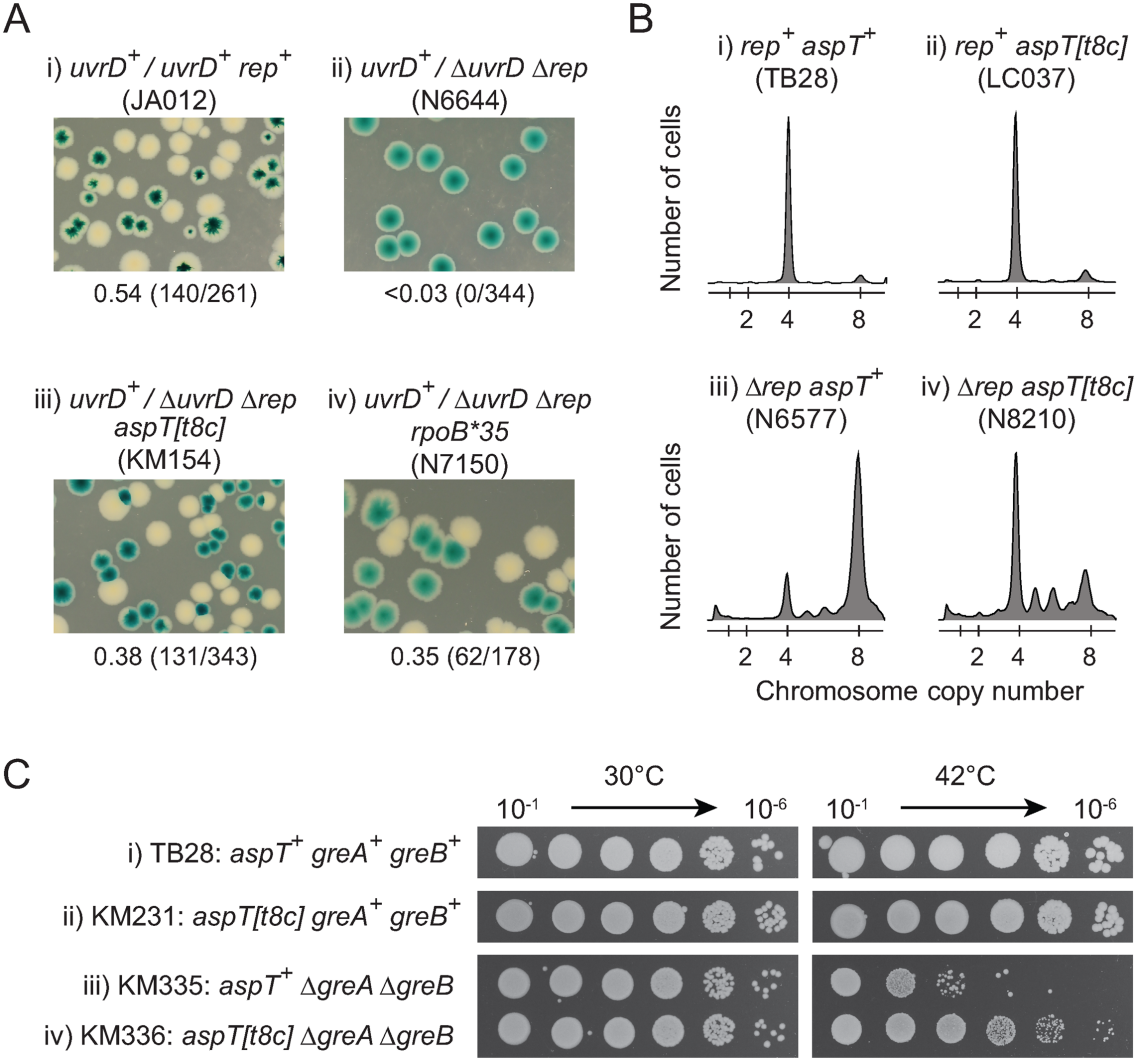
A mutation in an aspartyl tRNA gene suppresses the need for accessory replicative helicases and for anti-backtracking factors. (**A**) Retention or loss of pRC7*uvrD* (pAM407) from strains without or with Δ*rep* Δ*uvrD* deletions as judged by blue/white colony color on LB plates containing X-gal and IPTG. Fractions of white colonies are indicated below each image with actual numbers of white versus total colonies counted in parentheses. (**B**) DNA content of the indicated strains as monitored by flow cytometry under run out conditions in LB. The number of chromosome equivalents per cell is shown below. (**C**) The viability of *greA^+^ greB^+^* versus Δ*greA* Δ*greB* cells without and with *aspT[t8c]* as monitored by serial dilutions of liquid cultures grown at 30°C and plated subsequently on LB agar at 30°C and 42°C.

pRC7 is a very low copy plasmid which encodes β lactamase and can therefore be maintained in *E. coli* cells by the inclusion of ampicillin in the medium (65). However, the inefficient origin of replication within pRC7 results in rapid loss of the plasmid in the absence of ampicillin. This rapid loss can be detected if the host strain has a chromosomal deletion of *lacIZYA* since pRC7 encodes the *lac* operon and hence cells with and without the plasmid appear blue and white, respectively, on plates containing IPTG and X-gal (65). This retention or loss of pRC7 can be used to assess whether a gene is essential for colony formation by cloning of the test gene into pRC7, transformation of a Δ*lacIZYA* strain with the pRC7 derivative and subsequent deletion of the test gene from the chromosome. Plating of the strain onto medium containing IPTG and X-gal results in formation of only blue colonies if the test gene is essential or white and segregating colonies if the test gene is not essential (65). Rapid growth of *E. coli* requires accessory helicase activity and so pRC7 encoding *uvrD* can be lost rapidly from Δ*lacIZYA rep*^+^ *uvrD*^+^ cells on rich medium but pRC7*uvrD* cannot be lost from Δ*lacIZYA* Δ*rep* Δ*uvrD* cells as monitored by blue/white colony colour (34) (see also Figure 1A, compare i and ii). Spontaneous mutations that suppress this requirement for an accessory helicase can be isolated by exploiting the ability of Δ*rep* Δ*uvrD* cells to grow on minimal medium in the absence of a complementing pRC7 plasmid and subsequent plating of plasmid-less cells onto rich medium (34). Rare survivors on rich medium can then form colonies and the mutation(s) responsible for allowing Δ*rep* Δ*uvrD* cells to grow under rapid growth conditions can be analysed. We identified one such suppressor, the strain designation of which is N7182 (Supplementary Table S1). Potential linkage of the suppressor mutation to *rep* was tested by transducing Δ*rep*::*cat* from the suppressor strain into pRC7*uvrD/rep*^+^ *ΔuvrD::dhfr* (N6639). Eleven chloramphenicol-resistant transductants were tested for loss of pRC7*uvrD* on rich medium. Four transductants could not lose pRC7*uvrD* but seven could, indicating close linkage of the suppressor mutation with Δ*rep*::*cat* (see also Figure 1A, compare ii and iii).

We sequenced the genome of this suppressor strain and compared it with the genome of N7153, a Δ*rep* Δ*uvrD* strain that contains a well-characterized suppressor mutation rpoB*35 (5,10,34,66). Use of the Δ*rep* ΔuvrD rpoB*35 strain as a reference genome avoided the need for the reference strain to retain a complementing plasmid for viability. Only one mutation in N7182 that was not present in N7153 was located sufficiently close to Δ*rep::cat* to explain the above linkage (see Supplementary File 1). This mutation resulted in replacement of T with C at position 8 within the *aspT* gene, one of three identical tRNA^Asp^ genes in *E. coli*. Sequencing of the *aspT* gene from the 11 transductants obtained in the above cross revealed that all seven strains able to lose pRC7*uvrD* contained *aspT[t8c]* whereas all four that could not lose pRC7*uvrD* retained a wild type copy of *aspT*. Suppression of the inviability of Δ*rep* Δ*uvrD* cells on rich medium was therefore associated with the *aspT[t8c]* allele, with suppression comparable to that of rpoB*35 in a plasmid loss assay (Figure 1A, compare iii and iv).

Δ*rep uvrD*^+^ cells have a growth defect defect since UvrD can compensate only partially for the absence of Rep accessory helicase activity, resulting in slower movement of replication forks in Δ*rep* cells (39,40). Thus, the median number of copies of *oriC* in Δ*rep uvrD*^+^ cells is twice that of *rep*^+^ *uvrD*^+^ cells in rich medium due to an extended cell cycle in Δ*rep* cells and hence more replication initiation events per cell cycle (38). This doubling in *oriC* numbers results in a doubling of chromosome content when cells are treated with cephalexin and rifampicin to inhibit cell division and reinitiation of replication, so-called run-out conditions (38) (see also Figure 1B, compare i and iii). *aspT[t8c]* suppressed the increased chromosome copy number in Δ*rep uvrD*^+^ cells, reducing the median number of chromosomes from eight to four (Figure 1B, compare iii and iv). These data support the conclusion that this tRNA mutation reduces the need for accessory helicase activity. Furthermore, *aspT[t8c]* had no detectable impact on other UvrD-mediated processes. Defects in nucleotide excision repair, mismatch repair and control of recombination in *rep*^+^ Δ*uvrD* cells (52,67,68) were not suppressed by *aspT[t8c]* (Supplementary Figure S1A, B and C, respectively), providing further support for the specific suppression of the accessory helicase defect in Δ*rep* Δ*uvrD* cells by *aspT[t8c]*.

The requirement for accessory helicase activity is driven primarily by transcriptional barriers to replication (20,34). Backtracking of paused RNA polymerases results in formation of particularly stable replicative barriers and multiple factors have evolved to reduce the numbers of backtracked complexes (5). Cells lacking two homologous anti-backtracking factors, GreA and GreB, display a temperature-sensitive growth defect that is a consequence of more frequent collisions between replisomes and backtracked transcription complexes (5,10,69). *aspT[t8c]* suppressed the temperature-sensitive phenotype of Δ*greA* Δ*greB* cells (Figure 1C), similar to the suppression seen with rpoB*35 (48). We conclude that *aspT[t8c]* reduces the need for GreA/GreB-dependent rescue of backtracked RNA polymerase.

Taken together, these data indicate that *aspT[t8c]* reduces the need for Rep and UvrD to underpin replication and for anti-backtracking factors to resuscitate transcription complexes.

### Suppression by *aspT[t8c]* does not occur via growth rate restriction

The uridine encoded at position 8 within the wild type *aspT* gene is post-transcriptionally modified to 4-thiouridine and this modified nucleotide is conserved across all kingdoms of life (70). This residue is involved in a triple non-Watson–Crick pairing interaction and is important in coordination of magnesium within tRNA (71,72). This central structural role is reflected in the temperature-dependent destabilisation of human mitochondrial tRNA^Met^ structure by the same T to C mutation as found in *aspT[t8c]*. This transition mutation in tRNA^Met^ inhibits aminoacylation and any mutated tRNA^Met^ that is aminoacylated fails to form a stable ternary complex with elongation factor EF-Tu (70).

**Figure 2.**
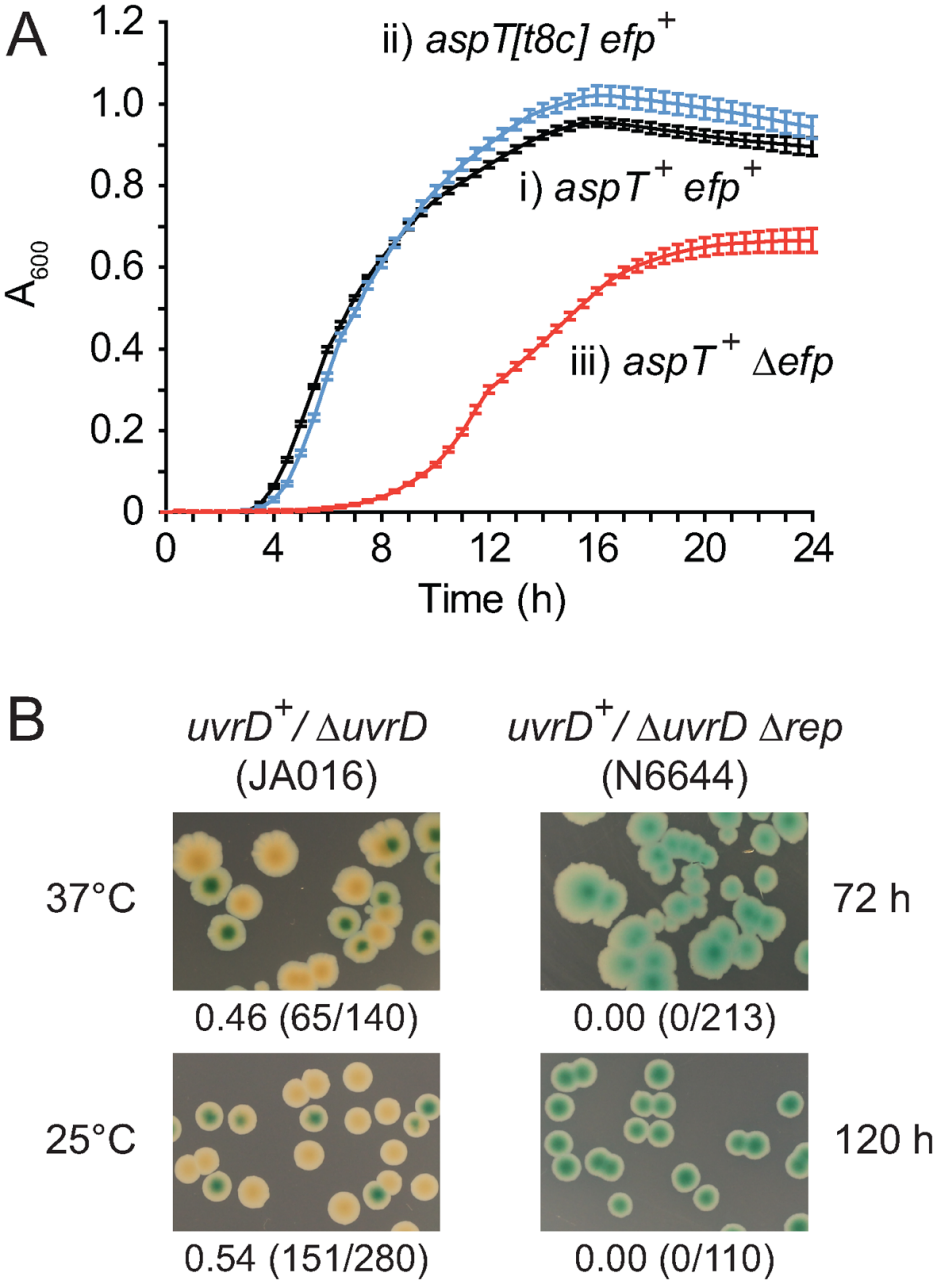
The impact of *aspT[t8c]* on growth and its relevance to Δ*rep* Δ*uvrD* inviability. (**A**) Growth of (i) *aspT*^+^*efp*^+^ (TB28), (ii) *aspT[t8c] efp*^+^ (KM231) and (iii) *aspT*^+^ Δ*efp* (MH299) in LB at 37°C as monitored by absorbance at 600 nm. (**B**) Assessment of the ability of pRC7*uvrD* (pAM407) to be lost from Δ*rep* Δ*uvrD* cells by reducing growth rates via culturing at 37°C and 25°C for the indicated times on defined rich medium containing glycerol as a carbon source.

Given the conservation of tRNA structure, *aspT[t8c]* may result in similar structural destabilisation of the encoded tRNA^Asp^ and consequent inhibition of interactions with aspartyl tRNA synthetase and EF-Tu. However, *aspT[t8c]* did not have a major impact on growth of *rep*^+^ *uvrD*^+^ cells in liquid culture (Figure 2A, compare i and ii). This absence of a significant growth defect in *aspT[t8c]* strains may be due to the presence of two other identical tRNA^Asp^ genes in *E. coli, aspU* and *aspV*.

Other means of growth restriction did not suppress Δ*rep* Δ*uvrD* inviability. Growth on defined rich medium containing all 20 amino acids but with a poor carbon source, glycerol, did not result in suppression of Δ*rep* Δ*uvrD aspT^+^* inviability at either 37°C or 25°C as determined using a plasmid loss assay (Figure 2B). Reduced growth rates therefore do not provide a general means of reducing the need for accessory helicase activity.

### Defective tRNA aminoacylation suppresses Δ*rep* Δ*uvrD* inviability

**Figure 3.**
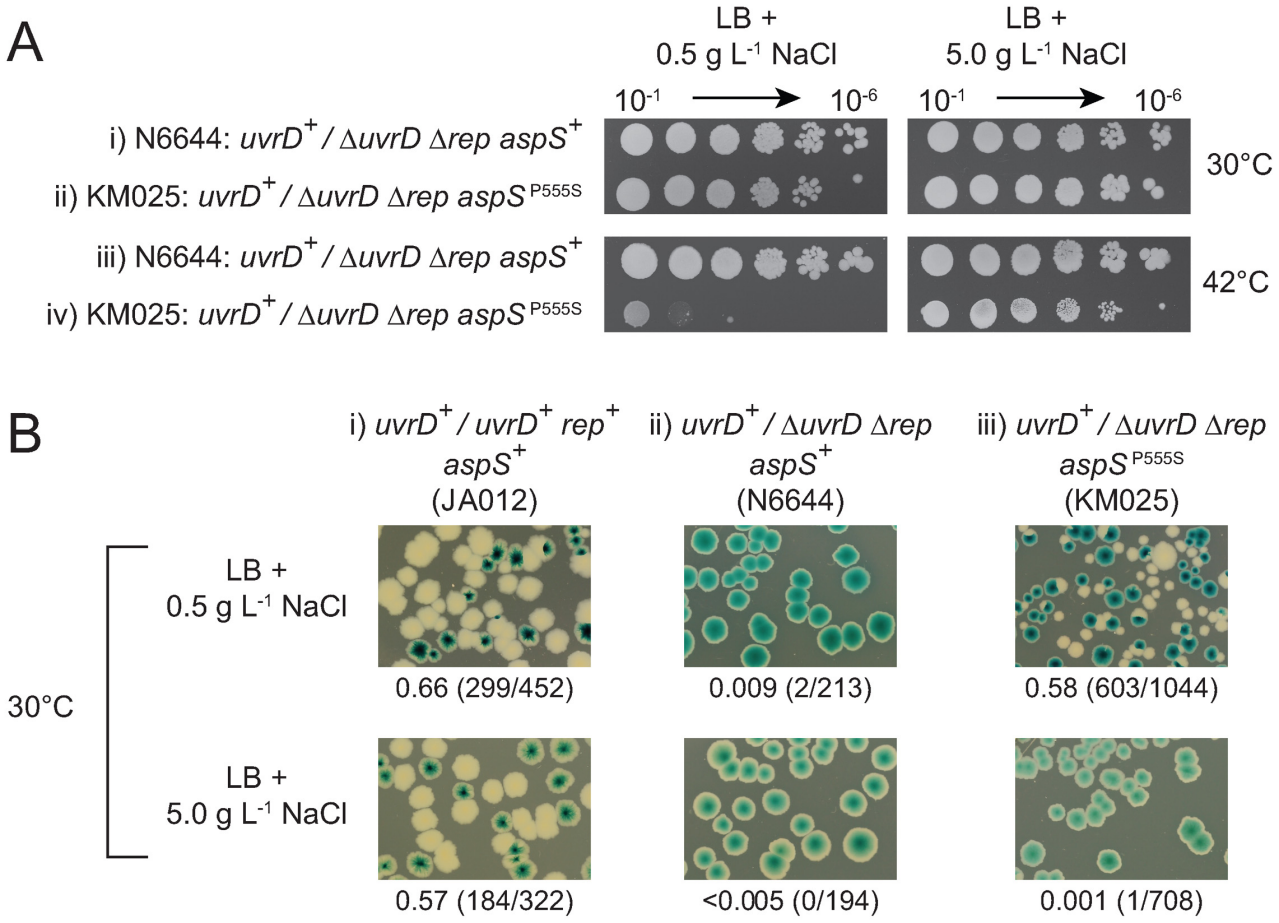
A mutation in aspartyl tRNA synthetase suppresses Δ*rep* Δ*uvrD* lethality. (**A**) The indicated strains were grown overnight in high salt medium at 30°C and then serial dilutions plated onto low and high salt plates containing 100 μg ml^−1^ ampicillin and incubated at either 30°C or 42°C. (**B**) Suppression of Δ*rep* Δ*uvrD* lethality by *aspS^P555S^* on rich medium was analysed by monitoring retention or loss of pRC7*uvrD* (pAM407) from the indicated strains at 30°C on either low or high salt LB medium.

As reduced growth rate was not the cause of suppression by *aspT[t8c]*, we probed whether defective translation was responsible. We could not detect significant defects in translation *in vivo* at tandem aspartate codons in an *aspT[t8c]* strain (Supplementary Figure S2). Either this allele does not result in translational pausing or such pausing is below the limits of detection using this assay. We therefore used alternative approaches to probe the *aspT[t8c]* suppression mechanism. The same t8c mutation in human mitochondrial tRNA^Met^ inhibits aminoacylation (70). Inhibition of tRNA^Asp^ aminoacylation was therefore tested for suppression of Δ*rep* Δ*uvrD* lethality. The *E. coli tls-1* allele encodes a P555S mutation in aspartyl tRNA synthetase that reduces the thermal stability of the synthetase and causes a severe reduction in growth rate at 42°C under low salt conditions (73,74) (see also Figure 3A). Given that AspRS^P555S^ is less stable than wild type enzyme regardless of the temperature (74), we tested whether *aspS^P555S^* suppressed Δ*rep* Δ*uvrD* lethality at 30°C on low salt medium, conditions under which viability is similar to wild type (73) (see also Figure 3A, compare i and ii). pRC7*uvrD* could be lost from Δ*rep* Δ*uvrD aspS^P555S^* at 30°C on low salt medium but not on high salt medium (Figure 3Biii). Suppression of Δ*rep* Δ*uvrD* lethality correlates therefore with a defect in aspartyl tRNA synthetase.

We also tested whether an alternative means of inhibiting tRNA^Asp^ aminoacylation could suppress Δ*rep* Δ*uvrD* inviability. A pRC7*uvrD*/Δ*rep* Δ*uvrD* strain defective in aspartate biosynthesis could lose the complementing pRC7*uvrD* plasmid on defined rich medium upon restriction of aspartate availability (Supplementary Figure S3). Thus a defect in the enzyme needed to synthesize aspartyl tRNA, AspRS, or a limiting concentration of one of the substrates needed for formation of aspartyl tRNA, aspartate, can suppress the need for accessory replicative helicase activity. Moreover, suppression was not specific to aspartate starvation as restriction of availability of phenylalanine also allowed Δ*rep* Δ*uvrD* cells to survive in the absence of a complementing plasmid (Supplementary Figure S4). As expected, restricting amino acid availability also resulted in very poor growth (Supplementary Figures S3 and S4) but a restricted growth rate does not by itself provide suppression of Δ*rep* Δ*uvrD* lethality (Figure 2B). These data support the conclusion that inhibition of aminoacylation of tRNA^Asp^, and of other tRNAs, reduces the need for accessory helicase activity.

### Translational pausing suppresses Δ*rep* Δ*uvrD* inviability

**Figure 4.**
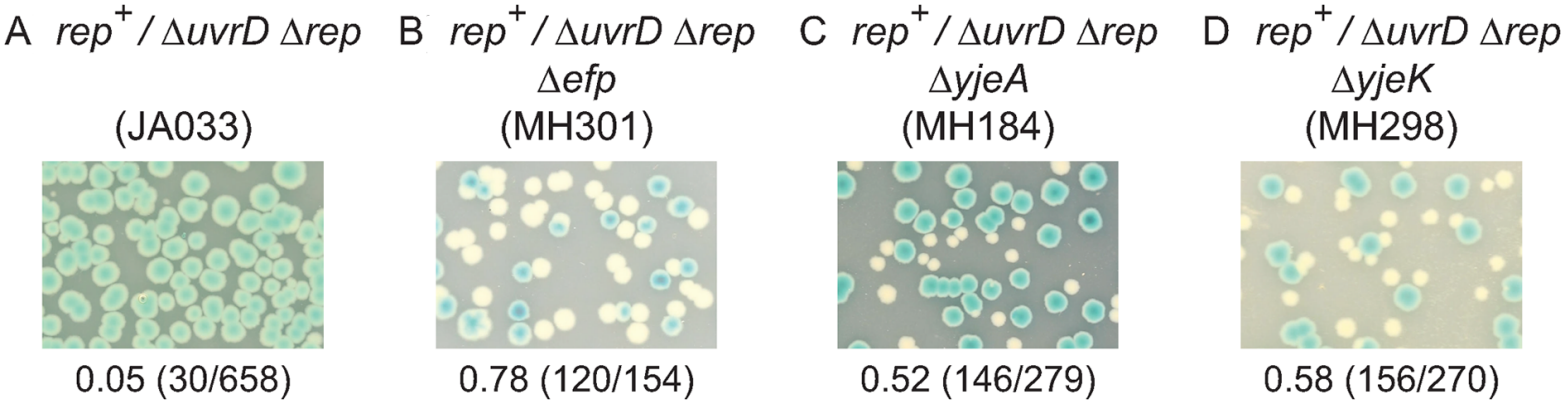
Ribosomal pausing decreases the requirement for accessory helicase activity. Retention or loss of pRC7*rep* (pAM403) was monitored at 37°C on LB X-gal IPTG plates for the indicated strains.

Suppression of Δ*rep* Δ*uvrD* inviability via inhibition of tRNA^Asp^ or tRNA^Phe^ aminoacylation might occur directly via pausing of ribosomes but could also occur via decreased production of one or more specific proteins containing both aspartate and phenylalanine. We tested therefore whether increased translational pausing by a well-defined mechanism that does not rely on decreased tRNA aminoacylation also suppresses the need for accessory helicase activity. Peptide bond formation by ribosomes occurs with low efficiency within polyproline tracts as compared with other amino acids and this low efficiency is compensated for by interaction of elongation factor P (EF-P) with the ribosome (56,75). When EF-P is absent (Δ*efp*) ribosomes pause for extended periods at tandem proline codons (56,75) (see also Supplementary Figure S2) resulting in reduced growth rates (76) (see also Figure 2A). Despite its negative effect on growth rate, introduction of Δ*efp* clearly suppressed Δ*rep* Δ*uvrD* lethality (Figure 4, compare A and B). *yjeA* and *yjeK* encode enzymes needed for post-translational modification of EF-P to form functional enzyme (56,75–78) and deletion of either *yjeA* or *yjeK* also resulted in suppression (Figure 4C and D). Enhancement of ribosomal pausing at polyproline sequences can therefore reduce the need for accessory helicase activity.

### Suppression by *aspT[t8c]* and Δ*efp* requires (p)ppGpp synthesis

One consequence of ribosomal pausing is increased synthesis of (p)ppGpp by RelA upon binding of RelA to a ribosome containing a non-acylated tRNA in the A site (13,14). Elevated (p)ppGpp is known to suppress Δ*rep* Δ*uvrD* inviability (34) and so *aspT[t8c]* and Δ*efp* might both therefore suppress via elevation of (p)ppGpp concentration.

**Figure 5.**
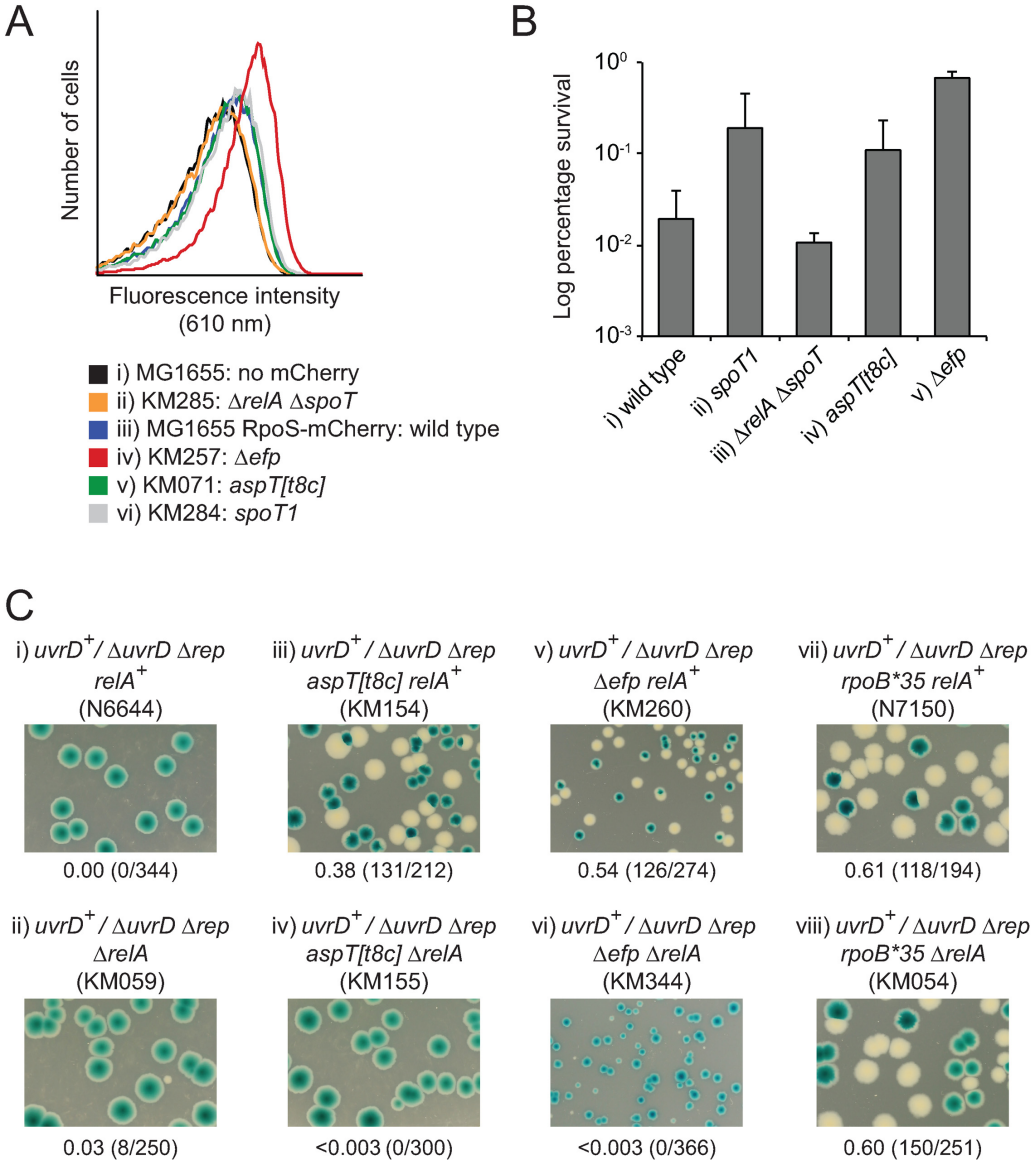
(p)ppGpp synthesis is needed for suppression of Δ*rep* Δ*uvrD* lethality by *aspT[t8c]* and Δ*efp*. (**A**) Flow cytometric detection of *in vivo* levels of RpoS-mCherry fluorescence in the indicated strains. All strains contain the *rpoS-mCherry* fusion with the exception of (i) MG1655. (**B**) Survival after challenge with ciprofloxacin. The strains are (i) TB28, (ii) KM241, (iii) N5777, (iv) KM231 and (v) MH372. (**C**) Retention and loss of pRC7*uvrD* (pAM407) on LB X-gal IPTG agar in *relA^+^* and Δ*relA* strains.

Direct measurement of (p)ppGpp using ^32^P cannot be performed on cells grown in rich media (79) which prevents direct assessment of (p)ppGpp levels in *aspT[t8c]* and Δ*efp* strains under conditions relevant to suppression of Δ*rep* Δ*uvrD* lethality. An indirect assay was therefore used that employs an RpoS-mCherry translational fusion (32). This reporter provides a fluorescence signal that correlates with intracellular (p)ppGpp concentration due to (p)ppGpp both stimulating *rpoS* transcription and inhibiting RpoS degradation (32). Fluorescence was assayed by flow cytometry of cells grown to mid-logarithmic phase in rich medium. *relA^+^ spoT^+^* cells lacking the RpoS–mCherry fusion and *rpoS-mCherry* Δ*relA* Δ*spoT* cells which are unable to synthesize (p)ppGpp gave similar levels of background fluorescence (Figure 5Ai and ii). An increase in fluorescence was observed in wild type cells carrying the fusion but this increase was modest (Figure 5A, compare iii with i and ii), consonant with low (p)ppGpp levels in wild type cells growing in nutrient-rich environments (32,80). mCherry fluorescence increased substantially in Δ*efp* cells but not in *aspT[t8c]* cells with respect to wild type (Figure 5A, compare iv and v with iii). We conclude that absence of EF-P results in elevation of (p)ppGpp concentration. Higher (p)ppGpp concentration in Δ*efp* cells as compared with wild type or *aspT[t8c]* cells is consistent with the significant retardation of growth of Δ*efp* cells (Figure 2A). In contrast, *aspT[t8c]* either does not increase (p)ppGpp levels as compared with wild type cells or any increase is below the limits of detection using this assay. To gauge the sensitivity of this assay we monitored fluorescence in *spoT1* cells. *spoT1* encodes a SpoT enzyme that retains (p)ppGpp synthase activity but lacks (p)ppGpp pyrophosphorylase activity, resulting in elevated (p)ppGpp (81) and the ability to suppress Δ*rep* Δ*uvrD* lethality (34). *spoT1 rpoS-mCherry* cells did not result in increased fluorescence as compared with *spoT^+^ rpoS-mCherry* cells indicating that (p)ppGpp levels sufficient to reduce the need for accessory replicative helicases could go undetected using this assay (Figure 5Aiii and vi).

To address this potential sensitivity problem we employed a second assay to determine whether either Δ*efp* or *aspT[t8c]* results in elevated (p)ppGpp. Cells with elevated (p)ppGpp display elevated levels of persistence and thus *spoT1* enhances whereas Δ*relA* Δ*spoT* alleles reduce persistence (32,82) (see also Figure 5Bi–iii). *aspT[t8c]* and Δ*efp* both increased persistence with the increase being higher for Δ*efp* (Figure 5Biv and v). The large increase in persistence in Δ*efp* cells correlates with the enhancement of fluorescence in Δ*efp rpoS-mCherry* cells. The level of persistence in *aspT[t8c]* cells is consistent with a smaller increase in (p)ppGpp levels as compared with Δ*efp* that is below the limits of detection in the mCherry fluorescence assay.

We tested whether increased (p)ppGpp contributed to suppression of Δ*rep* Δ*uvrD* inviability by *aspT[t8c]* and Δ*efp* by deleting the gene encoding the primary (p)ppGpp synthase, *relA*. Deletion of *relA* prevented loss of pRC7*uvrD* from Δ*rep* Δ*uvrD aspT[t8c]* and Δ*rep* Δ*uvrD* Δ*efp* cells, indicating that RelA was required for suppression by both mutant alleles (Figure 5C, compare iii with iv and v with vi). In contrast, pRC7*uvrD* could be lost from Δ*rep* Δ*uvrD* ΔrelA rpoB*35 cells indicating that RelA was not required for the viability of suppressed Δ*rep* Δ*uvrD* strains under all circumstances (Figure 5C, compare vii and viii). This lack of dependence of Δ*rep* ΔuvrD rpoB*35 on RelA likely reflects the recapitulation by rpoB*35 of many phenotypes associated with elevated (p)ppGpp even in the absence of RelA (48,66).

Taken together, these data indicate that RelA-directed synthesis of (p)ppGpp is important for suppression of Δ*rep* Δ*uvrD* lethality by both *aspT[t8c]* and Δ*efp*.

### *aspT[t8c]* and *Δefp* confer differing requirements for Rho activity

**Figure 6.**
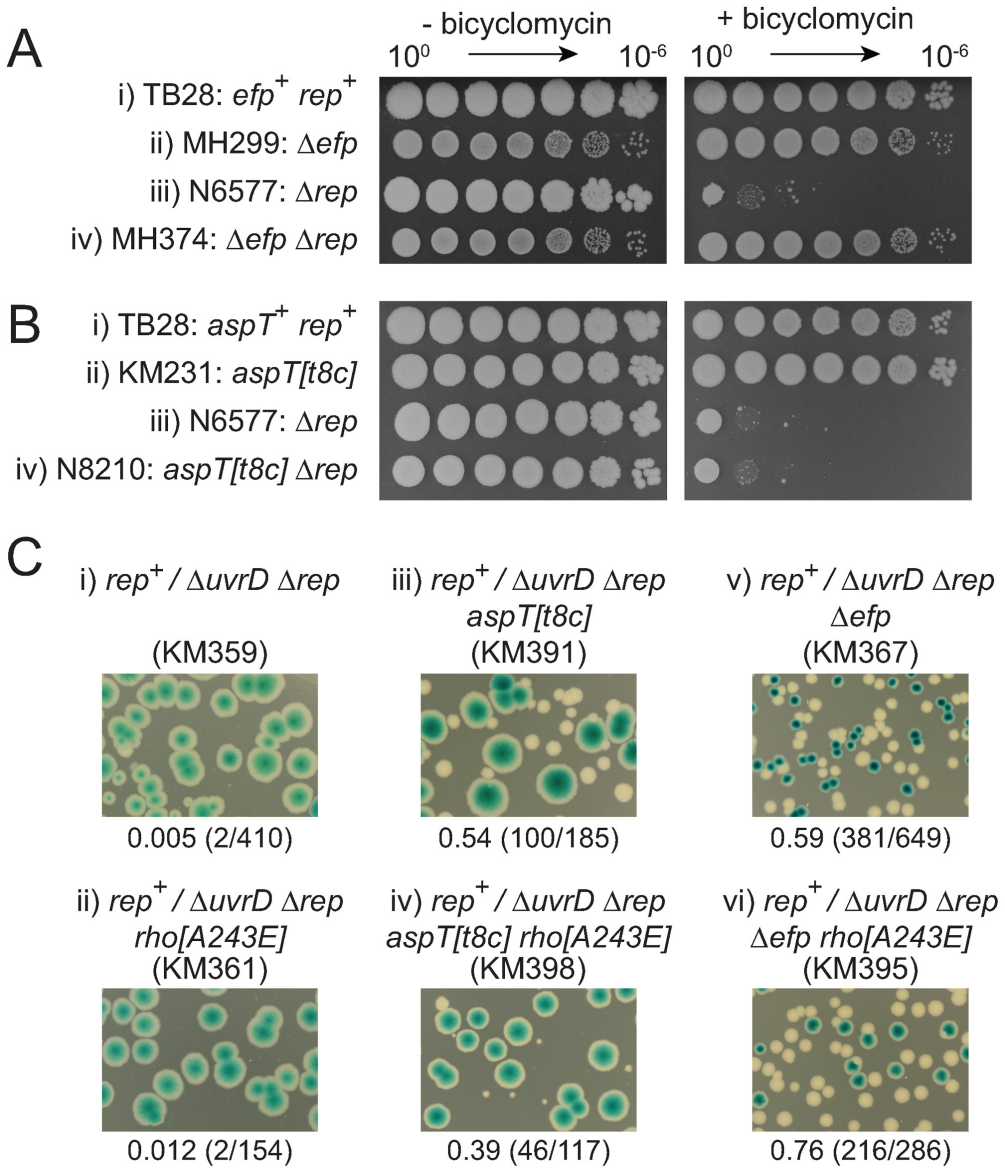
Δ*efp* but not *aspT[t8c]* can bypass the need for wild type Rho activity. (**A**) and (**B**) The indicated strains were grown in liquid culture in the absence of bicyclomycin and their ability to continue to divide with reduced Rho activity was assessed after serial dilution onto plates without and with 25 μg ml^−1^ bicyclomycin. (**C**) Loss of pRC7*rep* (pAM403) on LB X-gal IPTG agar in *rho*^+^ (i, iii, v) and *rho[A243E]* (ii, iv, vi) strains.

Translocation 5΄-3΄ by Rho along untranslated and unstructured nascent transcripts can displace transcription complexes thus reducing both RNA polymerase occupancy on the chromosome and R-loop formation (9,83,84). Rep and Rho therefore provide two different mechanisms that reduce the impact of transcription on replication. Consequently, cells need either Rep or wild type levels of Rho activity to maintain genome duplication in the face of transcriptional barriers (8,85). One manifestation of this requirement is the hypersensitivity of Δ*rep uvrD^+^* cells to low concentrations of the Rho-specific inhibitor bicyclomycin (8) (see also Figure 6A and B, compare i and iii).

We assessed whether Δ*efp* or *aspT[t8c]* could suppress Δ*rep* bicyclomycin hypersensitivity. Neither Δ*efp* nor *aspT[t8c]* altered bicyclomycin sensitivity in a *rep*^+^ background (Figure 6A and B, compare i and ii). In Δ*rep* cells hypersensitivity was suppressed by Δ*efp* but not by *aspT[t8c]* (Figure 6A and B, compare iii and iv). These data demonstrate that Δ*efp* but not *aspT[t8c]* can reduce the requirement for Rho activity in the absence of Rep.

We also tested whether this differential need for Rho activity was reflected in cells lacking both *rep* and *uvrD* by exploiting *rho[A243E]* which encodes a partial loss of function enzyme (86). Rho dependency was assessed by the ability of Δ*rep* Δ*uvrD* strains to lose a complementing pRC7*rep* rather than pRC7*uvrD* plasmid, allowing construction of pRC7*rep*/Δ*rep* Δ*uvrD rho[A243E]* strains regardless of *rep rho* double mutant lethality (85). The similar numbers and sizes of white plasmidless colonies formed by Δ*rep* Δ*uvrD* Δ*efp rho^+^* and Δ*rep* Δ*uvrD* Δ*efp rho[A243E]* cells indicate that wild type Rho function was not required for suppression by Δ*efp* (Figure 6C, compare v and vi). In contrast, Δ*rep* Δ*uvrD aspT[t8c] rho[A243E]* gave much smaller white plasmidless colonies as compared with the isogenic *rho^+^* strain indicating that wild type Rho function was important for growth of *aspT[t8c]* Δ*rep* Δ*uvrD* cells (Figure 6C, compare iii and iv).

**Figure 7.**
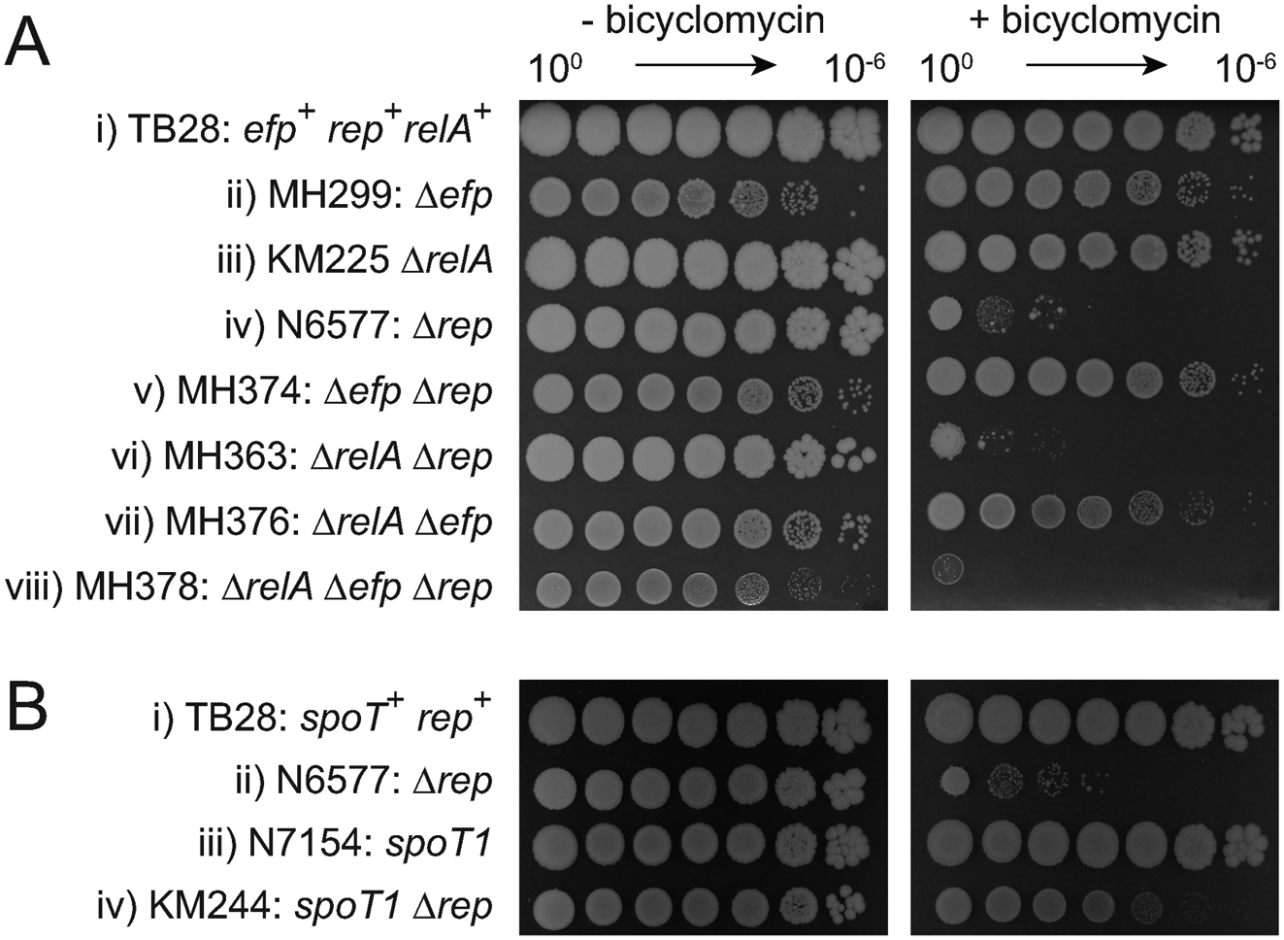
The ability of Δ*efp* to bypass the requirement for wild type Rho activity is dependent on (p)ppGpp synthesis. (**A**) and (**B**) Strains were grown in the absence of bicyclomycin and then serial dilutions were plated onto LB agar without and with 25 μg ml^−1^ bicyclomycin.

The data in Figures 5 and 6 indicate that the viability of *aspT[t8c]* Δ*rep* Δ*uvrD* cells requires both the major (p)ppGpp synthase in the cell, RelA, and wild type Rho activity. In contrast, Δ*efp* Δ*rep* Δ*uvrD* cells require RelA but not wild type Rho activity. Δ*efp* cells have higher levels of (p)ppGpp as compared with *aspT[t8c]* (Figure 5A) and these elevated levels might explain the differential requirement for Rho, given the ability of (p)ppGpp to reduce replication/transcription conflicts (10,34,66). Such a model implies that (p)ppGpp synthesis is critical not only for the viability of Δ*efp* Δ*rep* Δ*uvrD* cells (Figure 5C) but also for suppression of bicyclomycin sensitivity of Δ*rep* cells (Figure 6A). Absence of the primary (p)ppGpp synthase RelA did not hypersensitize otherwise wild type cells to bicyclomycin (Figure 7Aiii). However, the suppression of Δ*rep* bicyclomycin hypersensitivity by Δ*efp* was abolished upon deletion of *relA* (Figure 7A, compare viii with v). Thus RelA-dependent (p)ppGpp synthesis in Δ*rep* Δ*efp* cells is essential for survival with lowered Rho activity, supporting the hypothesis that elevated (p)ppGpp can reduce the need for Rho.

We tested this possible link between (p)ppGpp concentration and requirement for Rho by analysing the impact of other means of increasing (p)ppGpp concentration on Δ*rep* bicyclomycin hypersensitivity. The *spoT1* allele suppressed Δ*rep* bicyclomycin hypersensitivity supporting the conclusion that elevated (p)ppGpp can reduce the need for Rho (Figure 7B, compare iv with ii).

The data in Figures 5–7 indicate that both *aspT[t8c]* and Δ*efp* require RelA-directed synthesis of (p)ppGpp to maintain the viability of Δ*rep* Δ*uvrD* cells. In contrast, Δ*efp* has a much lower dependence on Rho activity as compared with *aspT[t8c]*. This differential requirement for Rho activity correlates with higher (p)ppGpp levels in Δ*efp* cells as compared with *aspT[t8c]*. Resolving conflicts between replication and transcription involves therefore a fine balance between accessory replicative helicases, levels of (p)ppGpp and Rho activity.

## DISCUSSION

We have discovered three new types of suppressors of Δ*rep* Δ*uvrD* rich medium lethality: mutations in tRNA genes, in aminoacyl-tRNA synthetases and in translation elongation factors. Whilst the mechanistic consequences of the *aspT[t8c]* mutation are unclear, the inhibition of tRNA aminoacylation by *aspS^P555S^* and of translation elongation by Δ*efp* are well-characterized (56,74,75), indicating that partial inhibition of translation can suppress Δ*rep* Δ*uvrD* lethality. Previous studies have demonstrated that this lethality is caused primarily by the conflict between replication and transcription (20,34,54). Thus partial inhibition of translation can compensate for the impact of transcription on DNA replication in the absence of Rep and UvrD. Suppression requires RelA-directed synthesis of (p)ppGpp with (p)ppGpp concentration being higher with Δ*efp* than with *aspT[t8c]* (Figure 5). Wild type levels of Rho activity are also needed in *aspT[t8c]* Δ*rep* Δ*uvrD* but not Δ*efp* Δ*rep* Δ*uvrD* cells indicating that the relative importance of (p)ppGpp and Rho function depends upon the nature of the translational mutation (Figure 6).

Δ*rep* Δ*uvrD* cells are inviable on rich medium primarily because of lack of accessory replicative helicase function (20,34,54). Suppression of this growth defect by the above translation mutations (Figures 1A, 3B and 4) therefore suggest that it is the lack of accessory helicase activity in Δ*rep* Δ*uvrD* cells that is being suppressed. Suppression by *aspT[t8c]* of the chromosome content defect of Δ*rep* cells, a direct consequence of slower fork movement in the absence of Rep accessory helicase activity (34,38,39), supports this view (Figure 1B). UvrD also inhibits RecFOR-dependent loading of RecA at blocked replication forks (52,68) and absence of this function makes a minor contribution to Δ*rep* Δ*uvrD* lethality (20,34,53). However, the lack of suppression of this RecA displacement defect by *aspT[t8c]* (Supplementary Figure S1C) indicates that suppression of Δ*rep* Δ*uvrD* lethality by *aspT[t8c]* does not operate via an effect on RecA loading. UvrD might also aid replication of transcribed DNA by inducing backtracking of RNAP stalled at DNA lesions, one consequence of which may be to promote repair of the lesion and allow the RNAP to then continue transcription (12). However, Δ*rep* Δ*uvrA* cells, lacking nucleotide excision repair, are viable (54) indicating that lesion repair via UvrD-catalysed backtracking of RNAP cannot be a major contributor to Δ*rep* Δ*uvrD* lethality. *aspT[t8c]* also does not suppress the sensitivity of Δ*uvrD* cells to UV light indicating that *aspT[t8c]* does not suppress nucleotide excision repair defects (Supplementary Figure S1A). Furthermore, *aspT[t8c]* suppression of the Δ*greA* Δ*greB* growth defect indicates that *aspT[t8c]* can suppress the absence of an *anti*-backtracking activity, making it difficult to explain how *aspT[t8c]* could also suppress the absence of a UvrD *pro*-backtracking function (Figure 1C).

RelA-directed synthesis of (p)ppGpp plays a central role in maintaining Δ*rep* Δ*uvrD* viability by *aspT[t8c]* and by Δ*efp* (Figure 5C). (p)ppGpp dramatically inhibits transcription of *rrn* operons (17) which are the primary transcriptional barriers to genome duplication (19,22), together with other highly expressed operons (18). (p)ppGpp may also destabilize stalled RNAP (10) or increase the fidelity of transcription (23), both of which could decrease the impact of transcription on fork movement. *aspT[t8c]*- and Δ*efp*-directed suppression of Δ*rep* Δ*uvrD* lethality via (p)ppGpp is therefore likely to occur by impacting on transcription initiation, stalled RNAP stability and/or decreased pausing due to enhanced fidelity.

RelA is stimulated to synthesize (p)ppGpp when an uncharged cognate tRNA is bound to the ribosomal A site (13,14). *aspT[t8c]* and Δ*efp* mutations may therefore increase the probability of A site-bound uncharged tRNA either directly or indirectly. The uridine at position 8 is highly conserved in tRNA species and the equivalent *t8c* mutation within human mitochondrial tRNA^Met^ results in inhibition of tRNA aminoacylation (70). If *aspT[t8c]* also resulted in inhibition of aminoacylation then the resulting increase in non-acylated tRNA^Asp^ could lead to an increased probability of uncharged tRNA^Asp^ occupying the ribosomal A site. However, whether the mutant tRNA^Asp^ can still bind to the A site is unknown. Alternatively tRNA*^asp[t8c]^* might poison the aspartyl tRNA synthetase by binding to it and forming a dead end complex with respect to aminoacylation. Formation of such a dead end complex might titrate out aspartyl tRNA synthetase and increase levels of uncharged wild type tRNA^Asp^ leading to increased non-acylated tRNA^Asp^ occupying the ribosomal A site. For cells lacking EF-P, YjeA or YjeK it is difficult to conceive how a reduced rate of proline–proline bond formation within the ribosome could lead directly to increased occupancy of the A site by uncharged tRNA. Absence of any one of these three factors, though, does impact on expression of many genes and so altered expression of one or more genes in Δ*efp*, Δ*yjeA* or Δ*yjeK* cells could result in an increased probability of A site-bound uncharged tRNA. For example, Δ*efp* cells have reduced expression of valyl tRNA synthetase (87) which could lead to accumulation of uncharged tRNA^Val^ and triggering of the stringent response. Suppression of Δ*rep* Δ*uvrD* lethality by *aspS^P555S^* demonstrates that suppression via partial loss of tRNA synthetase function can occur (Figure 3).

In contrast to the requirement for RelA to sustain suppression of Δ*rep* Δ*uvrD* lethality by both *aspT[t8c]* and Δ*efp*, the requirement for Rho activity is reduced in Δ*efp* as compared with *aspT[t8c]* cells (Figures 6 and 7). This difference correlates with the higher concentration of (p)ppGpp in Δ*efp* as compared with *aspT[t8c]* cells (Figure 5). A role for (p)ppGpp in reducing the need for Rho activity is supported by the ability of *spoT1* to suppress Δ*rep* bicyclomycin hypersensitivity (Figure 7B). Suppression of Δ*rep* bicyclomycin hypersensitivity by Δ*efp* also depends on RelA which supports a critical balance between (p)ppGpp concentration and Rho activity in maintaining viability (Figure 7A). Elevated (p)ppGpp can therefore reduce the need for Rho.

The greater dependence of *aspT[t8c]*-directed suppression on Rho might reflect not just lower (p)ppGpp levels in *aspT[t8c]* versus Δ*efp* cells but possibly also enhancement of Rho binding on nascent transcripts by *aspT[t8c]*. In other words, suppression via *aspT[t8c]* might occur at least partly via the increased generation of Rho binding sites on emerging transcripts. Aspartate codons are more frequent than polyproline tracts and hence *aspT[t8c]* has the potential to impact on ribosome translocation more frequently than Δ*efp*. Enhancement of Rho binding would also be dependent only on ribosomal pausing and not specifically require ribosomal A site occupancy by a non-aminoacylated tRNA (88), in contrast to stimulation of RelA activity (13,14). However, any *aspT[t8c]*-directed increase of transcription complex displacement by Rho cannot be sufficient by itself to suppress Δ*rep* Δ*uvrD* lethality since RelA is also needed (Figure 5C).

Our data indicate that whilst translation helps prevent RNAP backtracking (5,7) the reduction of conflicts between replication and transcription can be achieved more effectively by partial inhibition of translation. Of course, the growth defect in Δ*efp* cells (Figure 2A) indicates why the balance between replication, transcription, translation and (p)ppGpp synthesis is poised as it is in wild type cells. Thus for the maintenance of rapid growth there is a very fine balance to be struck between gene expression and accurate, rapid genome duplication. Furthermore, accessory replicative helicases play a major role in determining this balance. In the absence of both Rep and UvrD the wild type balance between various other mechanisms that reduce the impact of transcription on replication is unable to effectively counter the adverse effects of transcription on completion of genome duplication. There may also be circumstances under which mutations such as those identified here confer a selective advantage even in *rep^+^ uvrD^+^* cells. Elevated (p)ppGpp is a key factor in determining bacterial persistence in the face of antibiotic challenge (32) and both *aspT[t8c]* and Δ*efp* mutations enhance persistence (Figure 5B). It remains possible therefore that mutations such as *aspT[t8c]* that have only a modest inhibitory effect on growth could arise in bacterial populations continually exposed to antibiotics, especially given the many mutations known to affect translation (89). Increased persistence in strains lacking EF-P function also imply that EF-P and its unique post-translational modification pathway are poor potential targets for antibiotics.

## ACCESSION NUMBERS

Sequencing data were submitted in form of fastq files to the European Nucleotide Archive (ENA) and are available under accession number PRJEB14483 at http://www.ebi.ac.uk/ena/data/view/PRJEB14483.

## Supplementary Material

Supplementary Data

## References

[1] McGlynn P., Savery N.J., Dillingham M.S. The conflict between DNA replication and transcription. Mol. Microbiol. 2012; 85:12–20. 22607628 10.1111/j.1365-2958.2012.08102.x

[2] Hamperl S., Cimprich K.A. Conflict Resolution in the Genome: How Transcription and Replication Make It Work. Cell. 2016; 167:1455–1467. 27912056 10.1016/j.cell.2016.09.053 PMC5141617

[3] Landick R. The regulatory roles and mechanism of transcriptional pausing. Biochem. Soc. Trans. 2006; 34:1062–1066. 17073751 10.1042/BST0341062

[4] Komissarova N., Kashlev M. RNA polymerase switches between inactivated and activated states by translocating back and forth along the DNA and the RNA. J. Biol. Chem. 1997; 272:15329–15338. 9182561 10.1074/jbc.272.24.15329

[5] Dutta D., Shatalin K., Epshtein V., Gottesman M.E., Nudler E. Linking RNA polymerase backtracking to genome instability in *E. coli*.. Cell. 2011; 146:533–543. 21854980 10.1016/j.cell.2011.07.034 PMC3160732

[6] Epshtein V., Nudler E. Cooperation between RNA polymerase molecules in transcription elongation. Science. 2003; 300:801–805. 12730602 10.1126/science.1083219

[7] Proshkin S., Rahmouni A.R., Mironov A., Nudler E. Cooperation between translating ribosomes and RNA polymerase in transcription elongation. Science. 2010; 328:504–508. 20413502 10.1126/science.1184939 PMC2930199

[8] Washburn R.S., Gottesman M.E. Transcription termination maintains chromosome integrity. Proc. Natl. Acad. Sci. U.S.A. 2011; 108:792–797. 21183718 10.1073/pnas.1009564108 PMC3021005

[9] Krishna Leela J., Syeda A.H., Anupama K., Gowrishankar J. Rho-dependent transcription termination is essential to prevent excessive genome-wide R-loops in *Escherichia coli*. Proc. Natl. Acad. Sci. U.S.A. 2013; 110:258–263. 23251031 10.1073/pnas.1213123110 PMC3538224

[10] Trautinger B.W., Jaktaji R.P., Rusakova E., Lloyd R.G. RNA polymerase modulators and DNA repair activities resolve conflicts between DNA replication and transcription. Mol. Cell. 2005; 19:247–258. 16039593 10.1016/j.molcel.2005.06.004

[11] Orlova M., Newlands J., Das A., Goldfarb A., Borukhov S. Intrinsic transcript cleavage activity of RNA polymerase. Proc. Natl. Acad. Sci. U.S.A. 1995; 92:4596–4600. 7538676 10.1073/pnas.92.10.4596 PMC41991

[12] Epshtein V., Kamarthapu V., McGary K., Svetlov V., Ueberheide B., Proshkin S., Mironov A., Nudler E. UvrD facilitates DNA repair by pulling RNA polymerase backwards. Nature. 2014; 505:372–377. 24402227 10.1038/nature12928 PMC4471481

[13] Haseltine W.A., Block R. Synthesis of guanosine tetra- and pentaphosphate requires the presence of a codon-specific, uncharged transfer ribonucleic acid in the acceptor site of ribosomes. Proc. Natl. Acad. Sci. U.S.A. 1973; 70:1564–1568. 4576025 10.1073/pnas.70.5.1564 PMC433543

[14] Wendrich T.M., Blaha G., Wilson D.N., Marahiel M.A., Nierhaus K.H. Dissection of the mechanism for the stringent factor RelA. Mol. Cell. 2002; 10:779–788. 12419222 10.1016/s1097-2765(02)00656-1

[15] Potrykus K., Cashel M. (p)ppGpp: still magical?. Annu. Rev. Microbiol. 2008; 62:35–51. 18454629 10.1146/annurev.micro.62.081307.162903

[16] Battesti A., Bouveret E. Acyl carrier protein/SpoT interaction, the switch linking SpoT-dependent stress response to fatty acid metabolism. Mol. Microbiol. 2006; 62:1048–1063. 17078815 10.1111/j.1365-2958.2006.05442.x

[17] Barker M.M., Gaal T., Josaitis C.A., Gourse R.L. Mechanism of regulation of transcription initiation by ppGpp. I. Effects of ppGpp on transcription initiation *in vivo* and *in vitro*. J. Mol. Biol. 2001; 305:673–688. 11162084 10.1006/jmbi.2000.4327

[18] Durfee T., Hansen A.M., Zhi H., Blattner F.R., Jin D.J. Transcription profiling of the stringent response in *Escherichia coli*. J. Bacteriol. 2008; 190:1084–1096. 18039766 10.1128/JB.01092-07 PMC2223561

[19] Ton-Hoang B., Pasternak C., Siguier P., Guynet C., Hickman A.B., Dyda F., Sommer S., Chandler M. Single-stranded DNA transposition is coupled to host replication. Cell. 2010; 142:398–408. 20691900 10.1016/j.cell.2010.06.034 PMC2919506

[20] Boubakri H., de Septenville A.L., Viguera E., Michel B. The helicases DinG, Rep and UvrD cooperate to promote replication across transcription units *in vivo*. EMBO J. 2010; 29:145–157. 19851282 10.1038/emboj.2009.308 PMC2770101

[21] Srivatsan A., Tehranchi A., MacAlpine D.M., Wang J.D. Co-orientation of replication and transcription preserves genome integrity. PLoS Genet. 2010; 6:e1000810. 20090829 10.1371/journal.pgen.1000810 PMC2797598

[22] Merrikh H., Machon C., Grainger W.H., Grossman A.D., Soultanas P. Co-directional replication-transcription conflicts lead to replication restart. Nature. 2011; 470:554–557. 21350489 10.1038/nature09758 PMC3059490

[23] Roghanian M., Zenkin N., Yuzenkova Y. Bacterial global regulators DksA/ppGpp increase fidelity of transcription. Nucleic Acids Res. 2015; 43:1529–1536. 25605801 10.1093/nar/gkv003 PMC4330370

[24] Kamarthapu V., Epshtein V., Benjamin B., Proshkin S., Mironov A., Cashel M., Nudler E. ppGpp couples transcription to DNA repair in *E. coli*. Science. 2016; 352:993–996. 27199428 10.1126/science.aad6945 PMC4917784

[25] Wang J.D., Sanders G.M., Grossman A.D. Nutritional control of elongation of DNA replication by (p)ppGpp. Cell. 2007; 128:865–875. 17350574 10.1016/j.cell.2006.12.043 PMC1850998

[26] Maciag M., Kochanowska M., Lyzen R., Wegrzyn G., Szalewska-Palasz A. ppGpp inhibits the activity of *Escherichia coli* DnaG primase. Plasmid. 2010; 63:61–67. 19945481 10.1016/j.plasmid.2009.11.002

[27] Maciag-Dorszynska M., Szalewska-Palasz A., Wegrzyn G. Different effects of ppGpp on *Escherichia coli* DNA replication *in vivo* and *in vitro*. FEBS Open Biol. 2013; 3:161–164. 10.1016/j.fob.2013.03.001 PMC3668537 23772389

[28] Denapoli J., Tehranchi A.K., Wang J.D. Dose-dependent reduction of replication elongation rate by (p)ppGpp in *Escherichia coli* and *Bacillus subtilis*. Mol. Microbiol. 2013; 88:93–104. 23461544 10.1111/mmi.12172 PMC3640871

[29] Rojas A.M., Ehrenberg M., Andersson S.G., Kurland C.G. ppGpp inhibition of elongation factors Tu, G and Ts during polypeptide synthesis. Mol. Gen. Genet. 1984; 197:36–45. 6392824 10.1007/BF00327920

[30] Kanjee U., Ogata K., Houry W.A. Direct binding targets of the stringent response alarmone (p)ppGpp. Mol. Microbiol. 2012; 85:1029–1043. 22812515 10.1111/j.1365-2958.2012.08177.x

[31] Brauner A., Fridman O., Gefen O., Balaban N.Q. Distinguishing between resistance, tolerance and persistence to antibiotic treatment. Nat. Rev. Microbiol. 2016; 14:320–330. 27080241 10.1038/nrmicro.2016.34

[32] Maisonneuve E., Castro-Camargo M., Gerdes K. (p)ppGpp controls bacterial persistence by stochastic induction of toxin–antitoxin activity. Cell. 2013; 154:1140–1150. 23993101 10.1016/j.cell.2013.07.048

[33] McDonald K.R., Guise A.J., Pourbozorgi-Langroudi P., Cristea I.M., Zakian V.A., Capra J.A., Sabouri N. Pfh1 Is an Accessory Replicative Helicase that Interacts with the Replisome to Facilitate Fork Progression and Preserve Genome Integrity. PLoS Genet. 2016; 12:e1006238. 27611590 10.1371/journal.pgen.1006238 PMC5017727

[34] Guy C.P., Atkinson J., Gupta M.K., Mahdi A.A., Gwynn E.J., Rudolph C.J., Moon P.B., van Knippenberg I.C., Cadman C.J., Dillingham M.S. Rep provides a second motor at the replisome to promote duplication of protein-bound DNA. Mol. Cell. 2009; 36:654–666. 19941825 10.1016/j.molcel.2009.11.009 PMC2807033

[35] Bruning J.G., Howard J.L., McGlynn P. Accessory replicative helicases and the replication of protein-bound DNA. J. Mol. Biol. 2014; 426:3917–3928. 25308339 10.1016/j.jmb.2014.10.001

[36] Ivessa A.S., Lenzmeier B.A., Bessler J.B., Goudsouzian L.K., Schnakenberg S.L., Zakian V.A. The Saccharomyces cerevisiae helicase Rrm3p facilitates replication past nonhistone protein-DNA complexes. Mol. Cell. 2003; 12:1525–1536. 14690605 10.1016/s1097-2765(03)00456-8

[37] Azvolinsky A., Dunaway S., Torres J.Z., Bessler J.B., Zakian V.A. The S. cerevisiae Rrm3p DNA helicase moves with the replication fork and affects replication of all yeast chromosomes. Genes Dev. 2006; 20:3104–3116. 17114583 10.1101/gad.1478906 PMC1635146

[38] Gupta M.K., Guy C.P., Yeeles J.T., Atkinson J., Bell H., Lloyd R.G., Marians K.J., McGlynn P. Protein-DNA complexes are the primary sources of replication fork pausing in *Escherichia coli*. Proc. Natl. Acad. Sci. U.S.A. 2013; 110:7252–7257. 23589869 10.1073/pnas.1303890110 PMC3645559

[39] Lane H.E., Denhardt D.T. The rep mutation. IV. Slower movement of replication forks in *Escherichia coli rep* strains. J. Mol. Biol. 1975; 97:99–112. 1100854 10.1016/s0022-2836(75)80025-8

[40] Atkinson J., Gupta M.K., Rudolph C.J., Bell H., Lloyd R.G., McGlynn P. Localization of an accessory helicase at the replisome is critical in sustaining efficient genome duplication. Nucleic Acids Res. 2011; 39:949–957. 20923786 10.1093/nar/gkq889 PMC3035471

[41] Uzest M., Ehrlich S.D., Michel B. Lethality of *reprecB* and *rep recC* double mutants of *Escherichia coli*. Mol. Microbiol. 1995; 17:1177–1188. 8594336 10.1111/j.1365-2958.1995.mmi_17061177.x

[42] Michel B., Ehrlich S.D., Uzest M. DNA double-strand breaks caused by replication arrest. EMBO J. 1997; 16:430–438. 9029161 10.1093/emboj/16.2.430 PMC1169647

[43] Seigneur M., Bidnenko V., Ehrlich S.D., Michel B. RuvAB acts at arrested replication forks. Cell. 1998; 95:419–430. 9814711 10.1016/s0092-8674(00)81772-9

[44] Syeda A.H., Atkinson J., Lloyd R.G., McGlynn P. The balance between recombination enzymes and accessory replicative helicases in facilitating genome duplication. Genes. 2016; 7:E42. 27483323 10.3390/genes7080042 PMC4999830

[45] Atkinson J., Gupta M.K., McGlynn P. Interaction of Rep and DnaB on DNA. Nucleic Acids Res. 2011; 39:1351–1359. 20959294 10.1093/nar/gkq975 PMC3045612

[46] Taucher-Scholtz G., Abdel-Monem M., Hoffmann-Berling H. Cozzarelli NR Mechanisms of DNA Replication and Recombination. 1983; NY: Alan R. Liss Inc 65–76.

[47] Baharoglu Z., Lestini R., Duigou S., Michel B. RNA polymerase mutations that facilitate replication progression in the *rep uvrD recF* mutant lacking two accessory replicative helicases. Mol. Microbiol. 2010; 77:324–336. 20497334 10.1111/j.1365-2958.2010.07208.x PMC2936116

[48] Trautinger B.W., Lloyd R.G. Modulation of DNA repair by mutations flanking the DNA channel through RNA polymerase. EMBO J. 2002; 21:6944–6953. 12486015 10.1093/emboj/cdf654 PMC139083

[49] Nurse P., Zavitz K.H., Marians K.J. Inactivation of the *Escherichia coli priA* DNA replication protein induces the SOS response. J. Bacteriol. 1991; 173:6686–6693. 1938875 10.1128/jb.173.21.6686-6693.1991 PMC209016

[50] Mahdi A.A., Briggs G.S., Lloyd R.G. Modulation of DNA damage tolerance in *Escherichia coli recG* and *ruv*. strains by mutations affecting PriB, the ribosome and RNA polymerase. Mol. Microbiol. 2012; 86:675–691. 10.1111/mmi.12010 PMC3533792 22957744

[51] Syeda A.H., Hawkins M., McGlynn P. Recombination and replication. Cold Spring Harb. Perspect. Biol. 2014; 6:a016550. 25341919 10.1101/cshperspect.a016550 PMC4413237

[52] Veaute X., Delmas S., Selva M., Jeusset J., Le Cam E., Matic I., Fabre F., Petit M.A. UvrD helicase, unlike Rep helicase, dismantles RecA nucleoprotein filaments in *Escherichia coli*. EMBO J. 2005; 24:180–189. 15565170 10.1038/sj.emboj.7600485 PMC544901

[53] Petit M.A., Ehrlich D. Essential bacterial helicases that counteract the toxicity of recombination proteins. EMBO J. 2002; 21:3137–3147. 12065426 10.1093/emboj/cdf317 PMC126070

[54] Lestini R., Michel B. UvrD and UvrD252 counteract RecQ, RecJ, and RecFOR in a *rep* mutant of *Escherichia coli*. J. Bacteriol. 2008; 190:5995–6001. 18567657 10.1128/JB.00620-08 PMC2519539

[55] Mahdi A.A., Buckman C., Harris L., Lloyd R.G. Rep and PriA helicase activities prevent RecA from provoking unnecessary recombination during replication fork repair. Genes Dev. 2006; 20:2135–2147. 16882986 10.1101/gad.382306 PMC1536063

[56] Ude S., Lassak J., Starosta A.L., Kraxenberger T., Wilson D.N., Jung K. Translation elongation factor EF-P alleviates ribosome stalling at polyproline stretches. Science. 2013; 339:82–85. 23239623 10.1126/science.1228985

[57] Li H., Durbin R. Fast and accurate short read alignment with Burrows-Wheeler transform. Bioinformatics. 2009; 25:1754–1760. 19451168 10.1093/bioinformatics/btp324 PMC2705234

[58] Li H., Handsaker B., Wysoker A., Fennell T., Ruan J., Homer N., Marth G., Abecasis G., Durbin R. 1000 Genome Project Data Processing Subgroup The sequence alignment/map format and SAMtools. Bioinformatics. 2009; 25:2078–2079. 19505943 10.1093/bioinformatics/btp352 PMC2723002

[59] Thorvaldsdottir H., Robinson J.T., Mesirov J.P. Integrative Genomics Viewer (IGV): high-performance genomics data visualization and exploration. Brief. Bioinform. 2013; 14:178–192. 22517427 10.1093/bib/bbs017 PMC3603213

[60] Neidhardt F.C., Bloch P.L., Smith D.F. Culture medium for enterobacteria. J. Bacteriol. 1974; 119:736–747. 4604283 10.1128/jb.119.3.736-747.1974 PMC245675

[61] Low B. Rapid mapping of conditional and auxotrophic mutations in *Escherichia coli* K-12. J. Bacteriol. 1973; 113:798–812. 4570607 10.1128/jb.113.2.798-812.1973 PMC285295

[62] Lloyd R.G., Buckman C. Genetic analysis of the *recG* locus of *Escherichia coli* K-12 and of its role in recombination and DNA repair. J. Bacteriol. 1991; 173:1004–1011. 1846849 10.1128/jb.173.3.1004-1011.1991 PMC207218

[63] Theodore A., Lewis K., Vulic M. Tolerance of *Escherichia coli* to fluoroquinolone antibiotics depends on specific components of the SOS response pathway. Genetics. 2013; 195:1265–1276. 24077306 10.1534/genetics.113.152306 PMC3832272

[64] Hawkins M., Atkinson J., McGlynn P. *Escherichia coli* chromosome copy number measurement using flow cytometry analysis. Methods Mol. Biol. 2016; 1431:151–159. 27283308 10.1007/978-1-4939-3631-1_12

[65] Bernhardt T.G., de Boer P.A. Screening for synthetic lethal mutants in *Escherichia coli* and identification of EnvC (YibP) as a periplasmic septal ring factor with murein hydrolase activity. Mol. Microbiol. 2004; 52:1255–1269. 15165230 10.1111/j.1365-2958.2004.04063.x PMC4428336

[66] McGlynn P., Lloyd R.G. Modulation of RNA polymerase by (p)ppGpp reveals a RecG-dependent mechanism for replication fork progression. Cell. 2000; 101:35–45. 10778854 10.1016/S0092-8674(00)80621-2

[67] Friedberg E.C., Walker G.C., Siede W. Friedberg EC DNA Repair and Mutagenesis. 2006; 2nd edn, Washington, D.C: ASM Press.

[68] Magner D.B., Blankschien M.D., Lee J.A., Pennington J.M., Lupski J.R., Rosenberg S.M. RecQ promotes toxic recombination in cells lacking recombination intermediate-removal proteins. Mol. Cell. 2007; 26:273–286. 17466628 10.1016/j.molcel.2007.03.012 PMC2881834

[69] Sparkowski J., Das A. The nucleotide sequence of *greA*, a suppressor gene that restores growth of an *Escherichia coli* RNA polymerase mutant at high temperature. Nucleic Acids Res. 1990; 18:6443. 2243801 10.1093/nar/18.21.6443 PMC332557

[70] Jones C.N., Jones C.I., Graham W.D., Agris P.F., Spremulli L.L. A disease-causing point mutation in human mitochondrial tRNAMet rsults in tRNA misfolding leading to defects in translational initiation and elongation. J. Biol. Chem. 2008; 283:34445–34456. 18835817 10.1074/jbc.M806992200 PMC2590712

[71] Eiler S., Dock-Bregeon A., Moulinier L., Thierry J.C., Moras D. Synthesis of aspartyl-tRNA(Asp) in *Escherichia coli*–a snapshot of the second step. EMBO J. 1999; 18:6532–6541. 10562565 10.1093/emboj/18.22.6532 PMC1171716

[72] Jovine L., Djordjevic S., Rhodes D. The crystal structure of yeast phenylalanine tRNA at 2.0 A resolution: cleavage by Mg(2+) in 15-year old crystals. J. Mol. Biol. 2000; 301:401–414. 10926517 10.1006/jmbi.2000.3950

[73] Sharples G.J., Lloyd R.G. Location of a mutation in the aspartyl-tRNA synthetase gene of *Escherichia coli* K12. Mutat. Res. 1991; 264:93–96. 1944398 10.1016/0165-7992(91)90122-k

[74] Martin F., Sharples G.J., Lloyd R.G., Eiler S., Moras D., Gangloff J., Eriani G. Characterization of a thermosensitive *Escherichia coli* aspartyl-tRNA synthetase mutant. J. Bacteriol. 1997; 179:3691–3696. 9171418 10.1128/jb.179.11.3691-3696.1997 PMC179166

[75] Doerfel L.K., Wohlgemuth I., Kothe C., Peske F., Urlaub H., Rodnina M.V. EF-P is essential for rapid synthesis of proteins containing consecutive proline residues. Science. 2013; 339:85–88. 23239624 10.1126/science.1229017

[76] Yanagisawa T., Sumida T., Ishii R., Takemoto C., Yokoyama S. A paralog of lysyl-tRNA synthetase aminoacylates a conserved lysine residue in translation elongation factor P. Nat. Struct. Mol. Biol. 2010; 17:1136–1143. 20729861 10.1038/nsmb.1889

[77] Park J.H., Johansson H.E., Aoki H., Huang B.X., Kim H.Y., Ganoza M.C., Park M.H. Post-translational modification by beta-lysylation is required for activity of *Escherichia coli* elongation factor P (EF-P). J. Biol. Chem. 2012; 287:2579–2590. 22128152 10.1074/jbc.M111.309633 PMC3268417

[78] Navarre W.W., Zou S.B., Roy H., Xie J.L., Savchenko A., Singer A., Edvokimova E., Prost L.R., Kumar R., Ibba M. PoxA, YjeK, and elongation factor P coordinately modulate virulence and drug resistance in *Salmonella enterica*. Mol. Cell. 2010; 39:209–221. 20670890 10.1016/j.molcel.2010.06.021 PMC2913146

[79] Cashel M. Adolph KW Methods in Molecular Genetics. 1994; 3, NY: Academic Press 341–356.

[80] Ryals J., Little R., Bremer H. Control of rRNA and tRNA syntheses in *Escherichia coli* by guanosine tetraphosphate. J. Bacteriol. 1982; 151:1261–1268. 6179924 10.1128/jb.151.3.1261-1268.1982 PMC220404

[81] Laffler T., Gallant J.A. Stringent control of protein synthesis in *E. coli*. Cell. 1974; 3:47–49. 4607106 10.1016/0092-8674(74)90036-1

[82] Nguyen D., Joshi-Datar A., Lepine F., Bauerle E., Olakanmi O., Beer K., McKay G., Siehnel R., Schafhauser J., Wang Y. Active starvation responses mediate antibiotic tolerance in biofilms and nutrient-limited bacteria. Science. 2011; 334:982–986. 22096200 10.1126/science.1211037 PMC4046891

[83] Gaillard H., Aguilera A. Transcription as a threat to genome integrity. Annu. Rev. Biochem. 2016; 85:291–317. 27023844 10.1146/annurev-biochem-060815-014908

[84] Peters J.M., Vangeloff A.D., Landick R. Bacterial transcription terminators: the RNA 3΄-end chronicles. J. Mol. Biol. 2011; 412:793–813. 21439297 10.1016/j.jmb.2011.03.036 PMC3622210

[85] Fassler J.S., Tessman I., Tessman E.S. Lethality of the double mutations *rho rep* and *rho ssb* in *Escherichia coli*. J. Bacteriol. 1985; 161:609–614. 3155723 10.1128/jb.161.2.609-614.1985 PMC214926

[86] Harinarayanan R., Gowrishankar J. Host factor titration by chromosomal R-loops as a mechanism for runaway plasmid replication in transcription termination-defective mutants of *Escherichia coli*. J. Mol. Biol. 2003; 332:31–46. 12946345 10.1016/s0022-2836(03)00753-8

[87] Peil L., Starosta A.L., Lassak J., Atkinson G.C., Virumae K., Spitzer M., Tenson T., Jung K., Remme J., Wilson D.N. Distinct XPPX sequence motifs induce ribosome stalling, which is rescued by the translation elongation factor EF-P. Proc. Natl. Acad. Sci. U.S.A. 2013; 110:15265–15270. 24003132 10.1073/pnas.1310642110 PMC3780873

[88] Roberts J.W., Shankar S., Filter J.J. RNA polymerase elongation factors. Annu. Rev. Microbiol. 2008; 62:211–233. 18729732 10.1146/annurev.micro.61.080706.093422 PMC2819089

[89] Starosta A.L., Lassak J., Jung K., Wilson D.N. The bacterial translation stress response. FEMS Microbiol. Rev. 2014; 38:1172–1201. 25135187 10.1111/1574-6976.12083 PMC4227928

