# Supplementary material for: Inhibiting translation elongation can aid genome duplication in Escherichia coli
Source: Nucleic Acids Res. 2016 Dec 12;45(5):2571–84. doi: 10.1093/nar/gkw1254 (PMC5389703; doi:10.1093/nar/gkw1254)
Supplement: Supplementary Data [file gkw1254_Supp.zip › nar-02455-h-2016-File009.pdf]

Supplementary Information

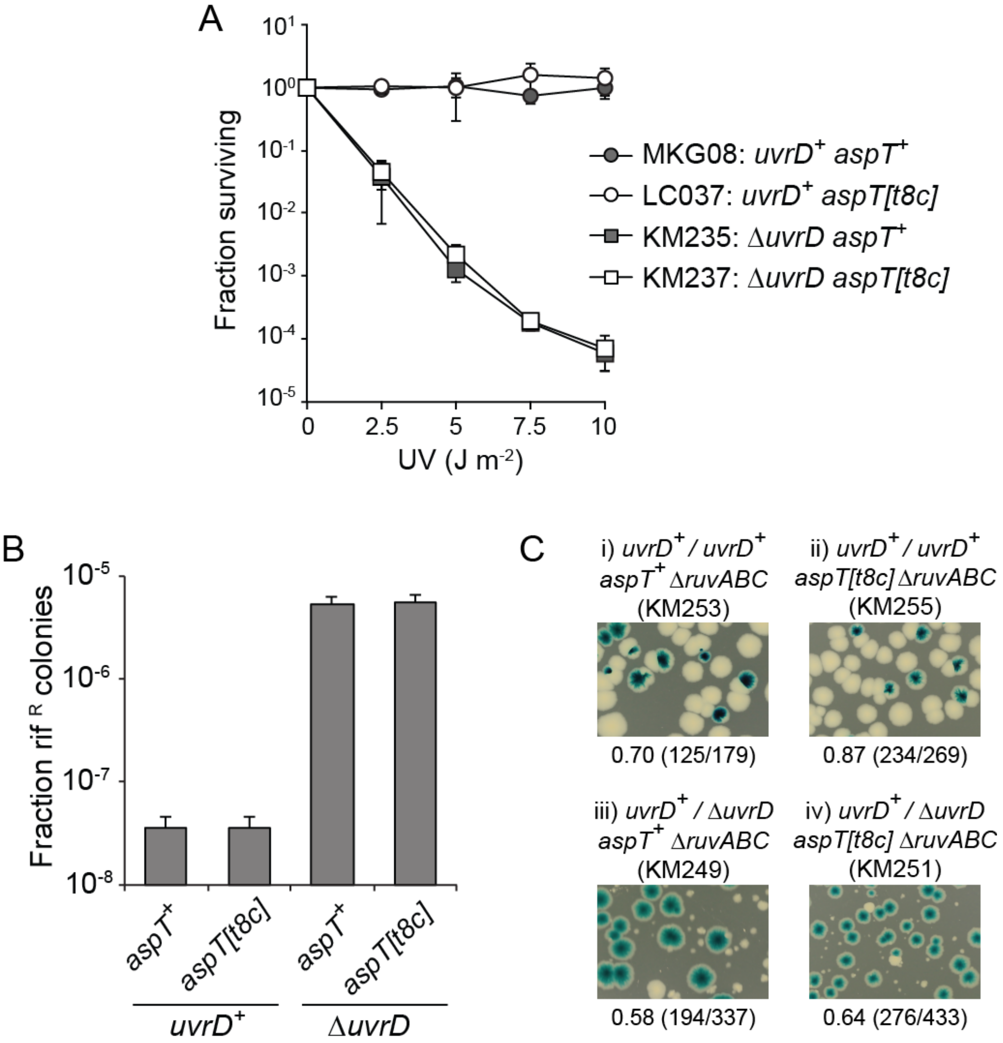

Supplementary Figure 1. *aspT[t8c]* does not suppress defects in nucleotide excision repair, mismatch repair or control of recombination in Δ*uvrD* cells.

(A) *Nucleotide excision repair.* The ability of the indicated strains to survive exposure to DNA damage was assessed at increasing doses of 254 nm UV light. Δ*uvrD* cells were sensitive to UV light as compared with *uvrD*$^+$ and this sensitivity was not suppressed by *aspT[t8c]* (compare KM235 and 237).

(B) *Mismatch repair.* The accumulation of rifampicin-resistant spontaneous mutations upon growth of a single colony to stationary phase in liquid culture was used as a readout of mismatch repair. The strains were those used in panel (A). Δ*uvrD* cells gave rise to more rifampicin resistant colonies as compared with *uvrD*$^+$ but this defect in mismatch repair was not suppressed by *aspT[t8c]*.

(C) *Control of recombination.* UvrD modulates levels of homologous recombination by displacing RecA from DNA (1). When RecA-directed strand exchange does proceed then RuvABC resolves any Holliday junctions formed thus facilitating chromosome segregation by removing interchromosomal Holliday junctions (2). Thus UvrD inhibits formation of interchromosomal Holliday junctions whilst RuvABC resolves such junctions when they do form. Efficient chromosome segregation requires either UvrD or RuvABC in *recA*$^+$ cells, evinced by the very low viability of Δ*uvrD* Δ*ruvABC* cells (2). Thus pRC7*uvrD* can be lost from *uvrD*$^+$ Δ*ruvABC* cells on LB agar at high frequency with no significant impact on plasmidless white colony size whereas plasmidless Δ*uvrD* Δ*ruvABC* colonies are very small, indicative of a severe viability problem (compare i with iii). This viability problem was not suppressed by *aspT[t8c]* (compare iii and iv).

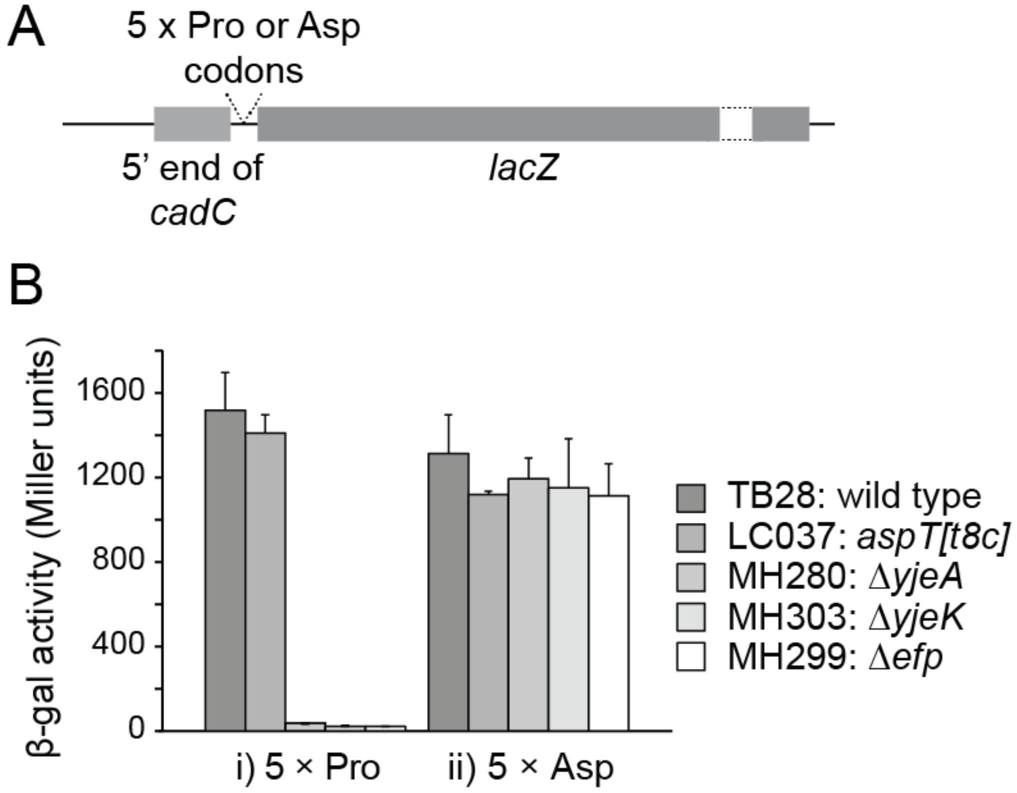

Supplementary Figure 2. *aspT[t8c]* does not cause significant pausing of ribosomes at aspartate codons.

(A) Schematic of the gene fusion cassette used to assess the relative impact of codons on efficiency of translation (3). The first 30 codons of *cadC* are fused in frame with *lacZ* via a linker sequence that encodes five consecutive aspartate or proline codons. β-galactosidase assays are then used with transformed *E. coli* cells to determine the impact of the repeated aspartate and proline codons on LacZ levels, providing a readout of translational efficiency.

(B) (i) YjeA, YjeK and EF-P are all needed for efficient translation of polyproline sequences and, as expected, levels of translation of the *lacZ* fusion protein from a plasmid containing five proline codons (p3LC-TL30-5P) (3) are inhibited when any one of these factors is absent as compared with a wild type strain. (ii) In contrast, the *aspT[t8c]* mutation does not result in significant inhibition of translation of *lacZ* from the plasmid encoding five tandem aspartate codons (p3LC-TL30-5D) as indicated by the similar levels of β-galactosidase activity in wild type and *aspT[t8c]* strains.

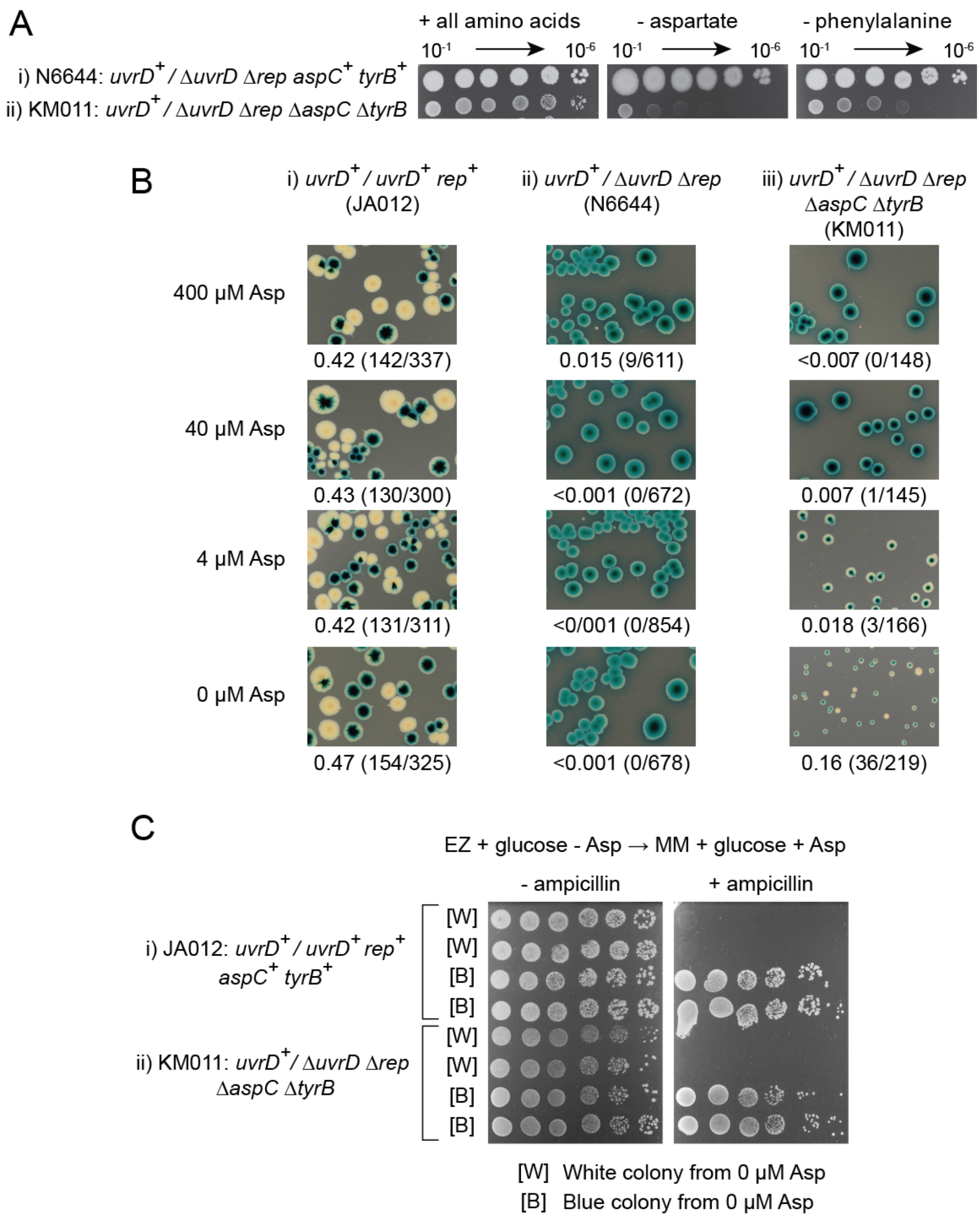

Supplementary Figure 3. Restriction of aspartate availability suppresses Δ*rep* Δ*uvrD* lethality.

(A) Aspartate auxotrophy requires mutations in both aspartate aminotransferase and aromatic amino acid aminotransferase, encoded by *aspC* and *tyrB* respectively, since each can catalyse the transamination of oxaloacetate from glutamate to form aspartate (4,5). Both these aminotransferases can also catalyse the final step in phenylalanine biosynthesis but *aspC tyrB* strains still synthesise phenylalanine since the branched chain amino acid aminotransferase encoded by *ilvE* can catalyse this reaction to a limited extent (4,6). We introduced both *aspC* and *tyrB* deletions into pRC7*uvrD*/Δ*rep* Δ*uvrD*. This strain (ii) could grow on defined rich medium when all amino acids were provided, although colony sizes were reduced. Absence of aspartate or phenylalanine exacerbated this growth defect severely but weak growth was still observable. Restricted growth was expected in the absence of phenylalanine via the *ilvE*-encoded branched chain amino acid aminotransferase (4,6). However, the

limited growth in the absence of added aspartate suggests that trace amounts of aspartate were present in preparations of the other amino acids present in the defined medium.

(B) Retention or loss of pRC7*uvrD* was monitored for the indicated strains on defined rich medium with decreasing concentrations of added aspartate. Absence of added aspartate had no significant impact on colony sizes or on frequency of loss of pRC7*uvrD* for either (i) JA012 or (ii) N6644, as expected since both strains retain the *aspC* and *tyrB* genes but colony sizes for (iii) KM011 decreased with decreasing aspartate concentration. Very small KM011 colonies could still form in the absence of added aspartate, consonant with the very restricted growth seen in (Aii), and some of these colonies were white indicating loss of pRC7*uvrD*. Limiting the availability of aspartate therefore suppresses Δ*rep* Δ*uvrD* lethality.

(C) Loss of pRC7*uvrD* from KM011 in the absence of added aspartate (see Biii) was confirmed by using ampicillin resistance as a marker for the presence or absence of pRC7*uvrD* which encodes β lactamase. Two white [W] and two blue [B] colonies of KM011 and JA012 from the 0 μM aspartate plates in (B) were resuspended in 100 μL 56/2 salts solution, serially diluted in the same salts solution and 5 μL of the 10^{0}-10^{-5} dilutions spotted onto minimal medium plates containing glucose and all amino acids without and with ampicillin. All dilutions of resuspended blue colonies gave colonies on plates regardless of the presence of ampicillin indicating that these colonies retained pRC7*uvrD* as expected. In contrast, all dilutions of resuspended white colonies gave colonies only in the absence of ampicillin. We conclude that the small white colonies isolated from KM011 on 0 μM aspartate plates in (B) had indeed lost pRC7*uvrD*, confirming suppression of Δ*rep* Δ*uvrD* lethality by restricting aspartate availability.

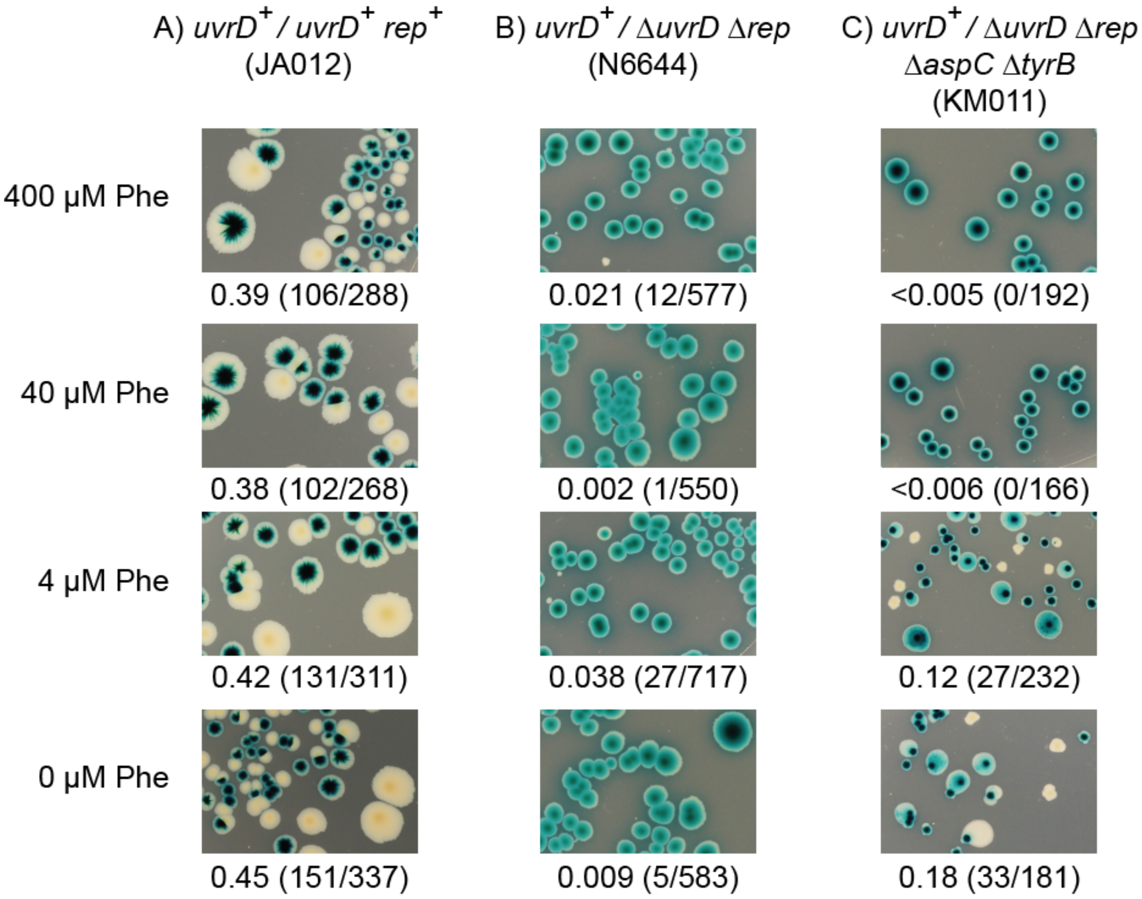

Supplementary Figure 4. Suppression of Δ*rep* Δ*uvrD* lethality by restriction of amino acid availability is not specific to aspartate metabolism.

The ability of a Δ*rep* Δ*uvrD* Δ*aspC* Δ*tyrB* strain, KM011, to lose pRC7*uvrD* at decreasing phenylalanine concentrations on defined rich medium was assessed. Colony morphology was altered at lower added phenylalanine concentrations but it was also clear that some of these colonies were white, indicating loss of pRC7*uvrD* (compare B and C at 4 and 0 µM phenylalanine). Restriction of phenylalanine availability therefore reduced the need for accessory replicative helicase activity in a manner similar to the suppression seen upon aspartate restriction (Supplementary Figure 3).

References

1. Veaute, X., Delmas, S., Selva, M., Jeusset, J., Le Cam, E., Matic, I., Fabre, F. and Petit, M.A. (2005) UvrD helicase, unlike Rep helicase, dismantles RecA nucleoprotein filaments in *Escherichia coli*. *EMBO J.*, **24**, 180-189.
2. Magner, D.B., Blankschien, M.D., Lee, J.A., Pennington, J.M., Lupski, J.R. and Rosenberg, S.M. (2007) RecQ promotes toxic recombination in cells lacking recombination intermediate-removal proteins. *Mol. Cell*, **26**, 273-286.
3. Ude, S., Lassak, J., Starosta, A.L., Kraxenberger, T., Wilson, D.N. and Jung, K. (2013) Translation elongation factor EF-P alleviates ribosome stalling at polyproline stretches. *Science*, **339**, 82-85.
4. Gelfand, D.H. and Steinberg, R.A. (1977) *Escherichia coli* mutants deficient in the aspartate and aromatic amino acid aminotransferases. *J. Bacteriol.*, **130**, 429-440.
5. Fotheringham, I.G., Dacey, S.A., Taylor, P.P., Smith, T.J., Hunter, M.G., Finlay, M.E., Primrose, S.B., Parker, D.M. and Edwards, R.M. (1986) The cloning and sequence analysis of the *aspC* and *tyrB* genes from *Escherichia coli* K12. Comparison of the primary structures of the aspartate aminotransferase and aromatic aminotransferase of *E. coli* with those of the pig aspartate aminotransferase isoenzymes. *Biochem. J.*, **234**, 593-604.
6. Lee-Peng, F.C., Hermodson, M.A. and Kohlhaw, G.B. (1979) Transaminase B from *Escherichia coli*: quaternary structure, amino-terminal sequence, substrate specificity, and absence of a separate valine-alpha-ketoglutarate activity. *J. Bacteriol.*, **139**, 339-345.

Supplementary Table 1. *Escherichia coli* K12 strains.

A) MG1655 derivatives

MG1655	F$^-$ *rph-1*	(1)
GJ6520	*lacI lacZU$_{118}$ trpR55 trpE9777 rho[A243E] Δ(yefM-yoeB)::Cm*	(2)
JA012	pAM407 *(lac$^+$ uvrD$^+$)* / *ΔlacIZYA::<>*	TB28 x pAM407 to Amp^r
JA016	pAM407 *(lac$^+$ uvrD$^+$)* / *ΔlacIZYA::<> ΔuvrD::dhfr*	N6632 x pAM407 to Amp^r
JA033	pAM403 *(lac$^+$ rep$^+$)* / *ΔlacIZYA::<> ΔuvrD::dhfr Δrep::cat*	Plasmid-free segregant of N6644 formed on minimal medium subsequently transformed with pAM403 to Amp^r
KM001	*ΔlacIZYA::<> ΔuvrD::dhfr ΔaspC745::<Kan>*	P1.JW0911 x N6632 to Km^r
KM003	*ΔlacIZYA::<> ΔuvrD::dhfr ΔaspC745::<>*	KM001 x pCP20 (3) to Amp^r then plasmid-free Km^s segregant identified after growth on LB at 42°C
KM005	*ΔlacIZYA::<> ΔuvrD::dhfr ΔaspC745::<> ΔtyrB747::<kan>*	P1.JW4014 x KM003 to Km^r
KM007	*ΔlacIZYA::<> ΔuvrD::dhfr ΔaspC745::<> ΔtyrB747::<>*	KM005 x pCP20 (3) to Amp^r then plasmid-free Km^s segregant identified after growth on LB at 42°C
KM009	pAM407 *(lac$^+$ uvrD$^+$)* / *ΔlacIZYA::<> ΔuvrD::dhfr ΔaspC745::<> ΔtyrB747::<>*	pAM407 x KM007 to Amp^r
KM011	pAM407 *(lac$^+$ uvrD$^+$)* / *ΔlacIZYA::<> ΔuvrD::dhfr Δrep::cat ΔaspC745::<> ΔtyrB747::<>*	P1.N6577x KM009 to Cm^r
KM021	pAM407 *(lac$^+$ uvrD$^+$)* / *ΔlacIZYA::<> ΔuvrD::dhfr tls-1 eda-51::*Tn*10*	P1.CS89 x N6632 to Tm^r
KM025	pAM407 *(lac$^+$ uvrD$^+$)* / *ΔlacIZYA::<> ΔuvrD::dhfr Δrep::cat tls-1 eda-51::*Tn*10*	P1.N6577 x KM021 to Cm^r
KM050	pAM407 *(lac$^+$ uvrD$^+$)* / *ΔlacIZYA::<> ΔuvrD::dhfr ΔrelA782::kan*	JA016 x P1.JW2755 to Km^r
KM054	pAM407 *(lac$^+$ uvrD$^+$)* / *ΔlacIZYA::<> rpoB*35 ΔuvrD::dhfr Δrep::cat ΔrelA782::kan*	N7150 x P1.JW2755 to Km^r
KM059	pAM407 *(lac$^+$ uvrD$^+$)* / *ΔlacIZYA::<> ΔuvrD::dhfr Δrep::cat ΔrelA782::kan*	N6644 x P1.JW2755 to Km^r
KM071	*rpoS-mCherry::<> rep$^+$<kan> aspT[t8c]*	MG1655 RpoS-Mcherry x P1.LC037 to Km^r
KM154	pAM407 *(lac$^+$ uvrD$^+$)* / *ΔlacIZYA::<> ΔuvrD::dhfr Δrep::cat aspT[t8c]*	JA016 x P1.PM647 to Cm^r. *aspT[t8c]* allele confirmed by sequencing
KM155	pAM407 *(lac$^+$ uvrD$^+$)* / *ΔlacIZYA::<> ΔuvrD::dhfr Δrep::cat aspT[t8c] ΔrelA782::kan*	KM050 x P1.N7182 to Cm^r
KM189	pAM407 *(lac$^+$ uvrD$^+$)* / *ΔlacIZYA::<> ΔuvrD::dhfr tls-1 Δeda775::kan*	KM021 x P1.JW1839 to Km^r and temperature-

		sensitive growth.
KM225	*ΔlacIZYA::<> ΔrelA782::kan*	TB28 x P1.JW2755 to Km^r
KM230	*lacI lacZU$_{118}$ trpR55 trpE9777 rho[A243E] Δ(yefM-yoeB)::Cm Δrep729::kan*	*Δrep729::kan* integration into GJ6520 using pKD46 (4)
KM231	*ΔlacIZYA::<> rep$^+$ <> aspT[t8c]*	LC037 x pCP20 (3) to Amp^r then plasmid-free Km^s segregant identified after growth on LB at 42°C
KM235	*ΔlacIZYA::<> ΔuvrD::dhfr rep$^+$ <kan>*	N6632 x P1.MKG08 to Km^r
KM237	*ΔlacIZYA::<> ΔuvrD::dhfr rep$^+$<kan> aspT[t8c]*	N6632 x P1.LC037 to Km^r
KM239	pAM407 *(lac$^+$ uvrD$^+$) / ΔlacIZYA::<> ΔuvrD::dhfr rep$^+$ <kan>*	KM235 x pAM407 to Amp^r
KM240	pAM407 *(lac$^+$ uvrD$^+$) / ΔlacIZYA::<> ΔuvrD::dhfr rep$^+$<kan> aspT[t8c]*	KM237 x pAM407 to Amp^r
KM241	*ΔlacIZYA::<> spoT1*	N7154 x pCP20 (3) to Amp^r then plasmid-free Km^s segregant identified after growth on LB at 42°C
KM244	*ΔlacIZYA::<> Δrep::cat spoT1*	PM567 x pCP20 (3) to Amp^r then plasmid-free Km^s segregant identified after growth on LB at 42°C
KM245	pAM407 *(lac$^+$ uvrD$^+$) / ΔlacIZYA::<> rep$^+$ <kan>*	MKG08 x pAM407 to Amp^r
KM246	pAM407 *(lac$^+$ uvrD$^+$) / ΔlacIZYA::<> rep$^+$<kan> aspT[t8c]*	LC037 x pAM407 to Amp^r
KM247	*ΔlacIZYA::<> spoT1 rpoS-mCherry::<kan>*	KM241 x P1. MG1655 RpoS-Mcherry Kan to Km^r
KM249	pAM407 *(lac$^+$ uvrD$^+$) / ΔlacIZYA::<> ΔuvrD::dhfr rep$^+$ <kan> ΔruvABC::cat*	KM239 x P1.N6268 to Cm^r
KM251	pAM407 *(lac$^+$ uvrD$^+$) / ΔlacIZYA::<> ΔuvrD::dhfr rep$^+$<kan> aspT[t8c] ΔruvABC::cat*	KM240 x P1.N6268 to Cm^r
KM253	pAM407 *(lac$^+$ uvrD$^+$) / ΔlacIZYA::<> rep$^+$ <kan> ΔruvABC::cat*	KM245 x P1.N6268 to Cm^r
KM255	pAM407 *(lac$^+$ uvrD$^+$) / ΔlacIZYA::<> rep$^+$<kan> aspT[t8c] ΔruvABC::cat*	KM246 x P1.N6268 to Cm^r
KM257	*rpoS-mCherry::<> Δefp772::kan*	MG1655 RpoS-Mcherry x P1.JW4107 to Km^r
KM260	pAM407 *(lac$^+$ uvrD$^+$) / ΔlacIZYA::<> ΔuvrD::dhfr Δrep::cat Δefp772::<>*	KM280 x P1.N6644 to Km^r
KM268	pAM407 *(lac$^+$ uvrD$^+$) / ΔlacIZYA::<> ΔuvrD::dhfr Δefp772::kan*	JA016 x P1.JW4107 to Km^r
KM269	pAM407 *(lac$^+$ uvrD$^+$) / ΔlacIZYA::<> rpoB*35 ΔuvrD::dhfr Δrep729::kan*	N7150 x P1.JW5604 to Km^r
KM271	pAM407 *(lac$^+$ uvrD$^+$) / ΔlacIZYA::<> rpoB*35 ΔuvrD::dhfr Δrep729::kan rho[A243E]*	N7150 x P1.KM230 to Km^r and then screening for the mutant *rho* allele by sequencing
KM273	pAM407 *(lac$^+$ uvrD$^+$) / ΔlacIZYA::<>*	N6644 x P1.KM269 to Km^r

	ΔuvrD::dhfr Δrep729::kan	
KM275	pAM407 (lac$^+$ uvrD$^+$) / ΔlacIZYA::<> Δefp772::<>	MH372 x pAM407 to Amp^r
KM277	pAM407 (lac$^+$ uvrD$^+$) / ΔlacIZYA::<> ΔuvrD::dhfr Δrep729::kan Δefp772::<>	KM275 x P1.KM269 to Km^r then screened for Tm^r
KM280	pAM407 (lac$^+$ uvrD$^+$) / ΔlacIZYA::<> ΔuvrD::dhfr Δefp772::<>	Plasmid-free segregant of KM268 formed on LB subsequently transformed with pCP20 (3) to Amp^r. Plasmid-free Km^s segregant then identified after growth on LB at 42°C and then retransformed with pAM407 to Amp^r.
KM281	pAM407 (lac$^+$ uvrD$^+$) / ΔlacIZYA::<> ΔuvrD::dhfr Δrep729::kan rho[A243E]	N6644 x P1.KM271 to Km^r
KM284	ΔlacIZYA::<> spoT1 rpoS-mCherry::<>	KM247 x pCP20 (3) to Amp^r then plasmid-free Km^s segregant identified after growth on LB at 42°C
KM285	rpoS-mCherry::<> ΔrelA251::kan ΔspoT207::cat	KM287 x P1.N5777 to Cm^r
KM287	rpoS-mCherry::<> ΔrelA251::kan	MG1655 RpoS-Mcherry x P1.N5777 to Km^r
KM301	pAM407 (lac$^+$ uvrD$^+$) / ΔlacIZYA::<> ΔuvrD::dhfr Δefp772::<> ΔrelA782::<>	MH376 x pCP20 (3) to Amp^r then plasmid-free Km^s segregant identified after growth on LB at 42°C. Km^s strain then transformed with pAM407 to Amp^r and transduced with P1.KM189 to Tm^r
KM307	pAM407 (lac$^+$ uvrD$^+$) / ΔlacIZYA::<> rep$^+$<> aspT[t8c]	KM231 x pAM407 to Amp^r
KM327	MG1655 ΔlacIZYA::<> ΔgreA788::<kan>	TB28 x P1.JW3148 to Km^r
KM331	ΔlacIZYA::<> rep$^+$<> aspT[t8c] ΔgreA788::<kan>	KM231 x P1.JW3148 to Km^r
KM335	MG1655 ΔlacIZYA::<> ΔgreA::<> ΔgreB740::<kan>	KM338 x P1.JW3369 to Km^r
KM336	ΔlacIZYA::<> rep$^+$<> aspT[t8c] ΔgreA788::<> ΔgreB740::<kan>	KM339 x P1.JW3369 to Km^r
KM337	pAM403 (lac$^+$ rep$^+$) / ΔlacIZYA::<> rep$^+$ <> aspT[t8c]	KM231 xpAM403 to Amp^r
KM338	ΔlacIZYA::<> ΔgreA788::<>	KM327 x pCP20 (3) to Amp^r then plasmid-free Km^s segregant identified after growth on LB at 42°C
KM339	ΔlacIZYA::<> rep$^+$<> aspT[t8c] ΔgreA788::<>	KM331 x pCP20 (3) to Amp^r then plasmid-free Km^s segregant identified after growth on LB at 42°C
KM344	pAM407 (lac$^+$ uvrD$^+$) / ΔlacIZYA::<> ΔuvrD::dhfr Δrep::cat Δefp772::<>	KM301 x P1.N6644 to Cm^r

	ΔrelA782::<>	
KM346	pAM407 *(lac^+ $uvrD^+$)* / *ΔlacIZYA::<> ΔuvrD::dhfr Δrep729::kan aspT[t8c] rho[A243E]*	KM307 x P1.KM271
KM359	pAM403 *(lac^+ rep^+)* / *ΔlacIZYA::<> ΔuvrD::dhfr Δrep729::kan*	Plasmid-free segregant of KM273 formed on minimal medium subsequently transformed with pAM403 to Amp^r
KM361	pAM403 *(lac^+ rep^+)* / *ΔlacIZYA::<> ΔuvrD::dhfr Δrep729::kan rho[A243E]*	Plasmid-free segregant of KM281 formed on minimal medium subsequently transformed with pAM403 to Amp^r
KM367	pAM403 *(lac^+ $uvrD^+$)* / *ΔlacIZYA::<> ΔuvrD::dhfr Δrep729::kan Δefp772::<>*	Plasmid-free segregant of KM277 formed on minimal medium subsequently transformed with pAM403 to Amp^r
KM389	pAM407 *(lac^+ $uvrD^+$)* / *ΔlacIZYA::<> ΔuvrD::dhfr Δrep729::kan aspT[t8c]*	KM154 x P1.KM346 to Km^r
KM391	pAM403 *(lac^+ rep^+)* / *ΔlacIZYA::<> ΔuvrD::dhfr Δrep729::kan aspT[t8c]*	Plasmid-free segregant of KM389 formed on minimal medium subsequently transformed with pAM403 to Amp^r
KM393	pAM403 *(lac^+ rep^+)* / *ΔlacIZYA::<> Δrep729::kan Δefp772::<> rho[A243E]*	MH388 x pAM403 to Amp^r
KM395	pAM403 *(lac^+ rep^+)* / *ΔlacIZYA::<> ΔuvrD::dhfr Δrep729::kan Δefp772::<> rho[A243E]*	KM393 x P1.KM271 to Tm^r
KM398	pAM403 *(lac^+ rep^+)* / *ΔlacIZYA::<> ΔuvrD::dhfr Δrep729::kan aspT[t8c] rho[A243E]*	KM337 x P1.KM346
LC037	*ΔlacIZYA::<> rep^+<kan> aspT[t8c]*	TB28 x P1.MKG121
MG1655 RpoS-Mcherry	*rpoS-mCherry::<>*	(5)
MG1655 RpoS-Mcherry Kan	*rpoS-mCherry::<kan>*	Kenn Gerdes
MH184	pAM403 *(lac^+ rep^+)* / *ΔlacIZYA::<> ΔuvrD::dhfr Δrep::cat ΔyjeA782::kan*	JA033 × P1.JW4116 to Km^r
MH280	*ΔlacIZYA::<> ΔyjeA782::kan*	TB28 × P1.JW4116 to Km^r
MH298	pAM403 *(lac^+ rep^+)* / *ΔlacIZYA::<> ΔuvrD::dhfr Δrep::cat ΔyjeK771::kan*	JA033 × P1.JW4106 to Km^r
MH299	*ΔlacIZYA::<> Δefp772::kan*	TB28 × P1.JW4107 to Km^r
MH300	*ΔlacIZYA::<> Δefp772::kan*	TB28 × P1.JW4107 to Km^r
MH301	pAM403 *(lac^+ rep^+)* / *ΔlacIZYA::<> ΔuvrD::dhfr Δrep::cat Δefp772::kan*	JA033 × P1.JW4107 to Km^r
MH303	*ΔlacIZYA::<> ΔyjeK771::kan*	TB28 × P1.JW4106 to Km^r
MH363	*ΔlacIZYA::<> Δrep::cat ΔrelA782::kan*	N6577 × P1. JW2755 to

		Km^r
MH364	*ΔlacIZYA::<> Δefp772::kan Δrep::cat*	MH300 × P1.PM412 to Cm^r
MH372	*ΔlacIZYA::<> Δefp772::<>*	MH299 x pCP20 (3) to Amp^r then plasmid-free Km^s segregant identified after growth on LB at 42°C
MH374	*ΔlacIZYA::<> Δefp772::<> Δrep::cat*	MH364 x pCP20 (3) to Amp^r then plasmid-free Km^s segregant identified after growth on LB at 42°C
MH376	*ΔlacIZYA::<> Δefp772::<> ΔrelA782::kan*	MH372 × P1. JW2755 to Km^r
MH378	*ΔlacIZYA::<> Δefp772::<> Δrep::cat ΔrelA782::kan*	MH374 × P1. JW2755 to Km^r
MH388	*ΔlacIZYA::<> Δrep729::kan Δefp772::<> rho[A243E]*	MH372 × P1.KM230 to Km^r then *rho* allele screened by sequencing
MKG08	*ΔlacIZYA::<>* rep^+ *<kan>*	(6)
MKG121	*ΔlacIZYA::<>* rep^+*<kan> aspT[t8c]*	rep^+ *<kan>* integration into N8210 using pKD46 (4)
N4304	*ΔrelA251::kan ΔspoT207::cat*	(7)
N5771	*ΔlacIZYA::<> ΔrelA251::kan*	TB28 x P1.N4304 to Km^r
N5777	*ΔlacIZYA::<> ΔrelA251::kan ΔspoT207::cat*	N5771 x P1.N4304 to Cm^r
N6268	*ΔlacIZYA::<> ΔruvABC::cat*	(8)
N6577	*ΔlacIZYA::<> Δrep::cat*	(9)
N6632	*ΔlacIZYA::<> ΔuvrD::dhfr*	(9)
N6639	pAM407 (lac^+ $uvrD^+$) / *ΔlacIZYA::<> ΔuvrD::dhfr*	(9)
N6644	pAM407 (lac^+ $uvrD^+$) / *ΔlacIZYA::<> ΔuvrD::dhfr Δrep::cat*	(9)
N7120	*ΔlacIZYA::<> ΔuvrD::dhfr Δrep::cat*	(9)
N7150	pAM407 (lac^+ $uvrD^+$) / *ΔlacIZYA::<> rpoB*35 ΔuvrD::dhfr Δrep::cat*	(9)
N7153	*ΔlacIZYA::<> rpoB*35 ΔuvrD::dhfr Δrep::cat*	Plasmid-free segregant of N7150
N7154	*ΔlacIZYA::<kan> spoT1*	(9)
N7182	*ΔlacIZYA::<> ΔuvrD::dhfr Δrep::cat aspT[t8c]*	Isolated as a larger colony variant from a culture of N7120 grown in 56/2 glucose medium and spread on LB agar
N7187	pAM407 (lac^+ $uvrD^+$) / *ΔlacIZYA::<kan> Δrep::cat spoT1*	(9)
N8210	*ΔlacIZYA::<> Δrep::cat aspT[t8c]*	P1.N7182 x TB28 to Cm^r
PM567	*ΔlacIZYA::<kan> Δrep::cat spoT1*	Plasmid-free segregant of N7187
TB28	*ΔlacIZYA::<>*	(10)

B) Other strains

BW25113	*rrnB3 ΔlacZ4787 hsdR514 Δ(araBAD)567 Δ(rhaBAD)568 rph-1*	(11)
JW0911	BW25113 *ΔaspC745::kan*	(11)
JW1839	BW25113 *Δeda775::kan*	(11)
JW2755	BW25113 *ΔrelA782::kan*	(11)
JW3148	BW25113 *ΔgreA788::kan*	(11)
JW3369	BW25113 *ΔgreB740::kan*	(11)
JW4014	BW25113 *ΔtyrB747747::kan*	(11)
JW4106	BW25113 *ΔyjeK771::kan*	(11)
JW4107	BW25113 *Δefp772::kan*	(11)
JW4116	BW25113 *ΔyjeA782::kan*	(11)
JW5604	BW25113 *Δrep729::kan*	(11)
AB1157	*araC14 thi-1 hisG4 Δ(gpt-proA)62 argE3 thr-1 leuB6 kdg51 rfbD1 lacY1 galK2 xyl-5 mtl-1 tsx-33 supE44 rac$^-$ mgl-51 rpsL31 qsr$^-$*	(1)
CS89	AB1157 *tls-1 eda-51::Tn10*	(12)

References

1. Bachmann, B.J. (1996) In Neidhardt, F. C., Curtiss III, R., Ingraham, J. L., Lin, E. C. C., Low, K. B., Magasanik, B., Reznikoff, W. S., Riley, M., Schaechter, M. and Umbarger, H. E. (eds.), *Escherichia coli and Salmonella cellular and molecular biology*. Second ed. ASM Press, Washington, DC, pp. 2460-2488.
2. Saxena, S. and Gowrishankar, J. (2011) Compromised factor-dependent transcription termination in a *nusA* mutant of *Escherichia coli*: spectrum of termination efficiencies generated by perturbations of Rho, NusG, NusA, and H-NS family proteins. *J. Bacteriol.*, **193**, 3842-3850.
3. Cherepanov, P.P. and Wackernagel, W. (1995) Gene disruption in *Escherichia coli*: Tc^R and Km^R cassettes with the option of Flp-catalyzed excision of the antibiotic-resistance determinant. *Gene*, **158**, 9-14.
4. Datsenko, K.A. and Wanner, B.L. (2000) One-step inactivation of chromosomal genes in *Escherichia coli* K-12 using PCR products. *Proc. Natl. Acad. Sci. U S A*, **97**, 6640-6645.
5. Maisonneuve, E., Castro-Camargo, M. and Gerdes, K. (2013) (p)ppGpp Controls Bacterial Persistence by Stochastic Induction of Toxin-Antitoxin Activity. *Cell*, **154**, 1140-1150.
6. Atkinson, J., Gupta, M.K., Rudolph, C.J., Bell, H., Lloyd, R.G. and McGlynn, P. (2011) Localization of an accessory helicase at the replisome is critical in sustaining efficient genome duplication. *Nucleic Acids Res.*, **39**, 949-957.
7. McGlynn, P. and Lloyd, R.G. (2000) Modulation of RNA polymerase by (p)ppGpp reveals a RecG-dependent mechanism for replication fork progression. *Cell*, **101**, 35-45.
8. Mahdi, A.A., Buckman, C., Harris, L. and Lloyd, R.G. (2006) Rep and PriA helicase activities prevent RecA from provoking unnecessary recombination during replication fork repair. *Genes Dev.*, **20**, 2135-2147.
9. Guy, C.P., Atkinson, J., Gupta, M.K., Mahdi, A.A., Gwynn, E.J., Rudolph, C.J., Moon, P.B., van Knippenberg, I.C., Cadman, C.J., Dillingham, M.S. *et al.* (2009) Rep Provides a Second Motor at the Replisome to Promote Duplication of Protein-Bound DNA. *Mol. Cell*, **36**, 654-666.
10. Bernhardt, T.G. and de Boer, P.A. (2004) Screening for synthetic lethal mutants in *Escherichia coli* and identification of EnvC (YibP) as a periplasmic septal ring factor with murein hydrolase activity. *Mol. Microbiol.*, **52**, 1255-1269.

11. Baba, T., Ara, T., Hasegawa, M., Takai, Y., Okumura, Y., Baba, M., Datsenko, K.A., Tomita, M., Wanner, B.L. and Mori, H. (2006) Construction of *Escherichia coli* K-12 in-frame, single-gene knockout mutants: the Keio collection. *Mol. Syst. Biol.*, **2**, 2006 0008.
12. Sharples, G.J. and Lloyd, R.G. (1991) Location of a mutation in the aspartyl-tRNA synthetase gene of *Escherichia coli* K12. *Mutat. Res.*, **264**, 93-96.
